# Flexible photonic contactless human-machine interface based on visible-blind near-infrared organic photodetectors

**DOI:** 10.1093/nsr/nwaf303

**Published:** 2025-07-26

**Authors:** Chen Geng, Guangkun Song, Wei Lin, Hanzhe Shi, Longyu Li, Zhaochen Suo, Yu Zhu, Hao Qin, Lin Liu, Ruiman Han, Yingjun Xia, Yanqing Yang, Tingting Guo, Xiangjian Wan, Bo Liu, Wangqiao Chen, Jing Zhang, Ting Zhang, Guanghui Li, Yongsheng Chen

**Affiliations:** The Centre of Nanoscale Science and Technology and Key Laboratory of Functional Polymer Materials, Institute of Polymer Chemistry, Renewable Energy Conversion and Storage Center (RECAST), College of Chemistry, Nankai University, Tianjin 300071, China; The Centre of Nanoscale Science and Technology and Key Laboratory of Functional Polymer Materials, Institute of Polymer Chemistry, Renewable Energy Conversion and Storage Center (RECAST), College of Chemistry, Nankai University, Tianjin 300071, China; Institute of Modern Optics, Tianjin Key Laboratory of Micro-scale Optical Information Science and Technology, Nankai University, Tianjin 300350, China; The Centre of Nanoscale Science and Technology and Key Laboratory of Functional Polymer Materials, Institute of Polymer Chemistry, Renewable Energy Conversion and Storage Center (RECAST), College of Chemistry, Nankai University, Tianjin 300071, China; The Centre of Nanoscale Science and Technology and Key Laboratory of Functional Polymer Materials, Institute of Polymer Chemistry, Renewable Energy Conversion and Storage Center (RECAST), College of Chemistry, Nankai University, Tianjin 300071, China; The Centre of Nanoscale Science and Technology and Key Laboratory of Functional Polymer Materials, Institute of Polymer Chemistry, Renewable Energy Conversion and Storage Center (RECAST), College of Chemistry, Nankai University, Tianjin 300071, China; The Centre of Nanoscale Science and Technology and Key Laboratory of Functional Polymer Materials, Institute of Polymer Chemistry, Renewable Energy Conversion and Storage Center (RECAST), College of Chemistry, Nankai University, Tianjin 300071, China; The Centre of Nanoscale Science and Technology and Key Laboratory of Functional Polymer Materials, Institute of Polymer Chemistry, Renewable Energy Conversion and Storage Center (RECAST), College of Chemistry, Nankai University, Tianjin 300071, China; i-Lab, Suzhou Institute of Nano-Tech and Nano-Bionics (SINANO), Chinese Academy of Sciences, Suzhou 215123, China; Guangdong Provincial Key Laboratory of Optical Information Materials and Technology, South China Academy of Advanced Optoelectronics, South China Normal University, Guangzhou 510006, China; The Centre of Nanoscale Science and Technology and Key Laboratory of Functional Polymer Materials, Institute of Polymer Chemistry, Renewable Energy Conversion and Storage Center (RECAST), College of Chemistry, Nankai University, Tianjin 300071, China; The Centre of Nanoscale Science and Technology and Key Laboratory of Functional Polymer Materials, Institute of Polymer Chemistry, Renewable Energy Conversion and Storage Center (RECAST), College of Chemistry, Nankai University, Tianjin 300071, China; The Centre of Nanoscale Science and Technology and Key Laboratory of Functional Polymer Materials, Institute of Polymer Chemistry, Renewable Energy Conversion and Storage Center (RECAST), College of Chemistry, Nankai University, Tianjin 300071, China; The Centre of Nanoscale Science and Technology and Key Laboratory of Functional Polymer Materials, Institute of Polymer Chemistry, Renewable Energy Conversion and Storage Center (RECAST), College of Chemistry, Nankai University, Tianjin 300071, China; Institute of Modern Optics, Tianjin Key Laboratory of Micro-scale Optical Information Science and Technology, Nankai University, Tianjin 300350, China; Guangdong Provincial Key Laboratory of Optical Information Materials and Technology, South China Academy of Advanced Optoelectronics, South China Normal University, Guangzhou 510006, China; The Centre of Nanoscale Science and Technology and Key Laboratory of Functional Polymer Materials, Institute of Polymer Chemistry, Renewable Energy Conversion and Storage Center (RECAST), College of Chemistry, Nankai University, Tianjin 300071, China; i-Lab, Suzhou Institute of Nano-Tech and Nano-Bionics (SINANO), Chinese Academy of Sciences, Suzhou 215123, China; The Centre of Nanoscale Science and Technology and Key Laboratory of Functional Polymer Materials, Institute of Polymer Chemistry, Renewable Energy Conversion and Storage Center (RECAST), College of Chemistry, Nankai University, Tianjin 300071, China; Academy for Advanced Interdisciplinary Studies, Nankai University, Tianjin 300071, China; The Centre of Nanoscale Science and Technology and Key Laboratory of Functional Polymer Materials, Institute of Polymer Chemistry, Renewable Energy Conversion and Storage Center (RECAST), College of Chemistry, Nankai University, Tianjin 300071, China; State Key Laboratory of Elemento-Organic Chemistry, Frontiers Science Center for New Organic Matter, College of Chemistry, Nankai University, Tianjin 300071, China

**Keywords:** flexible organic photodetector, visible-blind near-infrared photodetector, narrowband non-fullerene acceptor, contactless human-machine interface

## Abstract

Contactless human-machine interfaces (C-HMIs) are revolutionizing artificial intelligence (AI)-driven domains, yet face application limitations due to narrow sensing ranges, environmental fragility, and structural rigidity. To address these obstacles, we developed a flexible photonic C-HMI (Flex-PCI) using flexible visible-blind near-infrared organic photodetectors. In addition to its unprecedented performance across key metrics, including broad detection range (0.5–60.0 cm), high spatial resolution (∼10.0 µm), and fast response speed (1.6 µs), our Flex-PCI could precisely track finger kinematics and human physiological information, including position, velocity, trajectory, heart rate, and respiratory rate. Additionally, the Flex-PCI could stably operate under diverse conditions, such as various temperatures, humidities, ambient light intensities, bending states, and even underwater, addressing the reliability gap in dynamic applications. These combined, unparalleled characteristics have been demonstrated through a high-dimensional security system synergistically featuring tracking of finger kinematics and human physiological information in aerial and aquatic environments. This breakthrough technology opens up numerous possibilities for contactless interactions across diverse AI-powered scenarios, from security systems, social media, and AR/VR, to personal interactions with gaming and entertainment, significantly enhancing the quality of the user experience.

## Teaser

A flexible contactless HMI using a Vis-blind NIR OPD array tracks kinematics and physiological information with high speed, resolution, and robustness.

## INTRODUCTION

Realizing high-accuracy contactless interaction between humans and machines will have a transformative impact on artificial intelligence (AI)-powered technologies [1], such as virtual and augmented reality (AR/VR) [2], immersive games [3], automation equipment [4], and the rapidly evolving ‘Metaverse’ [5]. Contactless HMIs (C-HMIs) enable users to remotely interact with electronics through gestures in three-dimensional (3D) space, providing a more comfortable, convenient, user-friendly, and hygienic interaction between users and machines [6,7]. Beyond mere device control, C-HMI systems could also offer a more natural, realistic, intuitive, and immersive experience, bridging the gap between physical actions and virtual environments [8]. Despite significant advancements in C-HMIs based on various working mechanisms, such as humidity sensors [9,10], capacitive sensors [11,12], and thermal sensors [13,14], it remains challenging to concurrently and accurately perceive the high-dimensional information related to finger height, position, trajectory, and moving speed over a broad detection range. To be more specific, these sensors either exhibit poor sensitivity, limited detection range (<3 cm), or slow response/recovery speed (a few to dozens of seconds) exceeding the users’ recognition time (300–600 ms), leading to notable latency issues and an uncomfortable user experience [15–17]. Additionally, these systems frequently struggle with poor anti-interference capability to external stimuli from backgrounds such as heat, humidity, and electrical interferences, which causes erratic data and requires complex data-processing algorithms to extract accurate signals. These limitations severely hinder their practical applications in dynamic ambient conditions and underwater [18,19]. Camera-based optical C-HMIs, though widely used, often involve complex computing algorithms, calibration procedures, and privacy concerns [20]. Meanwhile, conventional photodetector-based optical systems, as a promising alternative, often suffer from intrinsic structural rigidities, bulkiness, and fixed band gaps, complicating their integration with state-of-the-art flexible electronics. Moreover, all these C-HMIs fail to precisely detect human vital signals due to their poor sensitivity or limited functionalities, significantly degrading users’ real experience in practical applications [20]. Consequently, when mapped onto a three-dimensional performance coordinate system, including performance, functionality, and anti-interference capability, all of the existing techniques lose one or multiple degrees of freedom, thus proving to be limiting in diversified ambient environments and underwater (Table S1 in the online Supplementary file).

To fully realize the potential of C-HMIs in cutting-edge electronics and provide a more intuitive, realistic, and immersive user experience, it is essential to develop high-dimensional devices capable of accurately capturing detailed gesture information, including basic kinematics and physiological information under diverse conditions [20]. Therefore, C-HMIs should simultaneously exhibit high performance (such as high sensitivity, fast response speed, and long-distance detection capability), robust stability under varying conditions, and multifunctionality, together with flexible structure and low-power consumption [21]. Unfortunately, to the best of our knowledge, no existing technologies can simultaneously meet these ideal performance criteria, primarily due to the lack of suitable high-performance devices and the absence of design principles for contactless systems.

Solution-processed thin-film near-infrared (NIR) organic photodetectors (OPDs) offer distinct superiorities over conventional inorganic photodetectors [22–24], such as intrinsic flexibility [25,26], tailorable detection peak [27–29], tunable semitransparency [30], and shape/size versatility, making them promising candidates for flexible C-HMIs. However, it remains challenging to fabricate flexible NIR OPDs that simultaneously exhibit high sensitivity, fast response, and high selectivity for specific NIR light, such as the 850 nm light that is safe for the human eye and strongly reflected by human skin [31]. Moreover, the lack of established design principles for OPD-based flexible C-HMIs further hinders their development and applications in contactless systems [16,32].

**Figure 1.**
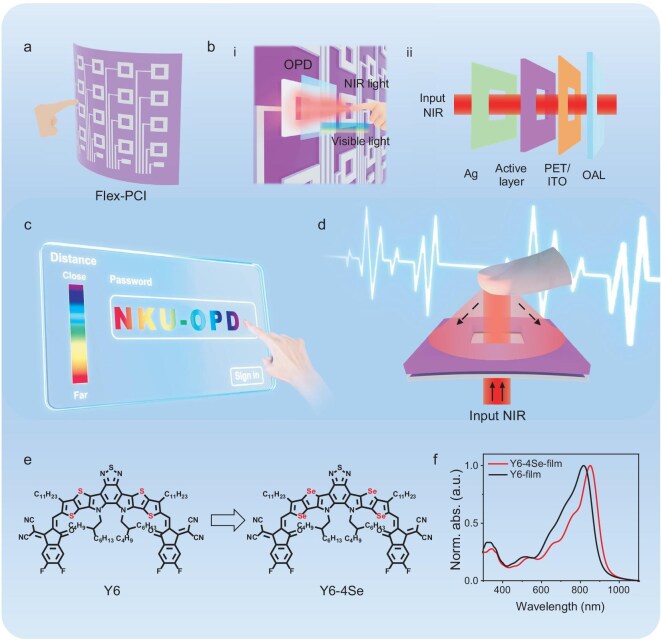
The Flex-PCI based on flexible Vis-blind NIR OPDs. (a) Schematic diagram of Flex-PCI based on a flexible OPD array. (b) Working mechanism (i) and exploded views of the Flex-PCI (ii). The contactless detection of finger kinematics (position, height, trajectory, and moving speed) (c), and human physiological information (heart rate and respiratory rate) (d) using Flex-PCI. (e) The chemical structure of Y6 and Y6-4Se. (f) The normalized solution and thin film absorption profiles of Y6 and Y6-4Se.

Here, we present a comprehensive strategy encompassing dedicatedly designed materials, optimized device architecture, theoretical simulation, and compact system layout to realize such a flexible photonic C-HMI (Flex-PCI) based on NIR OPD. The flexible NIR OPD exhibits a strong visible light anti-interference capability with a peak responsivity of 0.34 A W^−^^1^ and a response time of 580 ns at 850 nm. Then, we designed and fabricated a unique Flex-PCI, featuring a finely tuned OPD array (4.1 pixels per inch, ppi) with a hollow structure (Fig. 1a and b) to enhance sensitivity and improve long-range detection. Such a design successfully realized real-time and concurrent tracking of finger position, height, trajectory, moving speed, heart rate, and respiratory rate (Fig. 1c and d). The Flex-PCI achieves unprecedented performance across key metrics, including a broad detection range (0.5–60.0 cm), high spatial resolution (∼10 µm), and fast response speed (1.6 µs) in diverse ambient conditions and even underwater, effectively addressing the performance and stability issues faced by current HMIs. As a demonstration of its unprecedented and combined characteristics, we designed a high-dimensional contactless security system that simultaneously detects finger kinematics and human physiological information, offering a breakthrough in secure, seamless, and hygienic human-machine interaction. With its exceptional performance, intrinsic flexibility, multifunctionality, and stability, the innovative Flex-PCI provides new opportunities for contactless interaction with next-generation AI-powered devices, particularly in security systems, social media, AR/VR, and personal interactions with gaming and entertainment.

## RESULTS AND DISCUSSION

### Optimization of materials and devices

The high-performance non-fullerene acceptor (NFA) Y6 has a highly favorable three-dimensional (3D) molecular packing network, which facilitates efficient charge transport, and has been broadly utilized to fabricate high-performance OPDs [33]. However, the OPD with Y6 NFA shows weak responsivity in the desired NIR region (∼850 nm) and relatively slow response, making it unsuitable for the Flex-PCI [33]. To address these limitations, we introduced a modified NFA, Y6-4Se, by incorporating highly polarizable selenium (Se) atoms into the benzothiadiazole core of star acceptor Y6 (Fig. 1e) [34]. This atomic-level modification aims to realize three key objectives: (1) enhancing the quinoidal resonance character and redshifting the peak absorption to ∼850 nm by leveraging the large and loose outermost electron cloud of Se [34]; (2) maintaining the favorable 3D molecular packing network of Y6 to the maximum and strengthening the intermolecular Se-Se interactions to improve charge mobility and increase the response speed of OPD [35–38]; and (3) increasing molecular rigidity and improving packing to reduce trap states and energetic disorders and enhance the sensitivity of the OPD [39]. As depicted in Fig. 1f, the Y6-4Se film indeed shows a significant redshift of 30 nm in the absorption onset compared to the Y6 film, with a peak absorption at 850 nm, indicating substantial potential in detecting NIR light (especially 850 nm) for the high-sensitivity Flex-PCI. Detailed characterizations and discussions of Y6-4Se, Y6 acceptors, and PM6 polymer donors are presented in Figs S1 and S2, Table S2, and Texts S1, S2 in the online Supplementary file.

To suppress the noise current, here we fabricated NIR OPD using an inverted device architecture, where MoO_x_ and ZnO serve as the hole and electron transporting layers, respectively (Fig. S3a and Text S3) [40]. As shown in Fig. S4 and Table S3, the hole/electron charge mobilities of PM6:Y6-4Se blend films is 5.49/6.01 × 10^−^^4^ cm^−^^2^ V^−^^1^ s^−^^1^, which is higher than the value of the PM6:Y6 blend of 5.10/5.44 × 10^−^^4^ cm^−^^2^ V^−^^1^ s^−^^1^. Moreover, the transient photocurrent (TPC) measurement, along with the analysis of the dependencies of open-circuit voltage (${V}_{oc}$) and short-circuit current density (${J}_{sc}$) on light intensity (${P}_{\textit{light}}$), collectively validates the enhanced charge transport and effective suppression of charge accumulation and recombination in OPD based on PM6:Y6-4Se (Figs S5, S6, and Text S4). The device with PM6:Y6-4Se blend shows a trap density (*N_A_*) of 4.14 × 10^15^ cm^−^^3^ and Urbach Energy (*E_u_*) of 17.09 meV, much lower than the corresponding values of the device based on PM6:Y6 (*N_A_* = 4.04 × 10^15^ cm^−^^3^, *E_u_* = 18.01 meV), which can efficiently suppress noise current generation (Fig. S7, Tables S4 and S5) [41]. Such enhanced performance is ascribed to the stronger and more ordered molecular packing and fibrillar network interpenetrating structure in the PM6:Y6-4Se film (Figs S8, S9, and Table S6). Detailed characterizations and discussions of OPD performance and film morphology are presented in Text S4. Consequently, the optimized NIR OPD with PM6:Y6-4Se blend demonstrates a lower noise current (2.88 × 10^–14^ A Hz^-1/2^), higher responsivity (0.43 A W^−^^1^), and faster response speed (474 ns) compared to corresponding values of the device with PM6:Y6 blend at 850 nm (Fig. S10 and Table S7).

### Design, fabrication, and characterizations of flexible Vis-blind NIR OPDs

**Figure 2.**
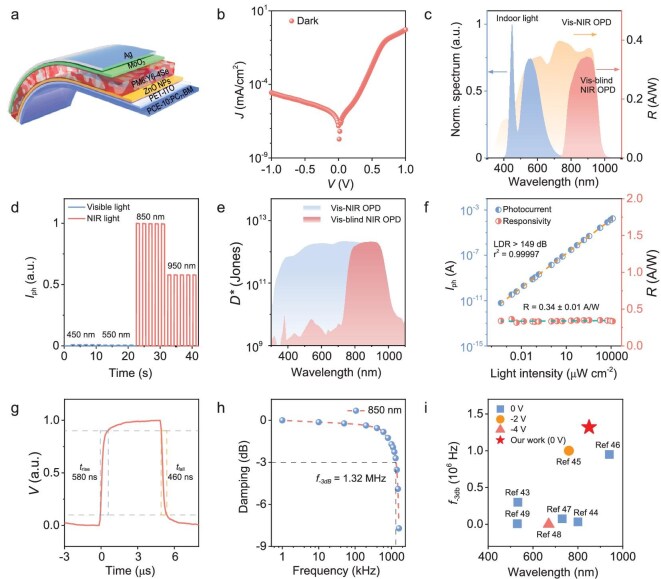
Optoelectronic performance of flexible Vis-blind NIR OPD based on PM6:Y6-4Se blend. (a) Device structure of the flexible Vis-blind NIR OPD. (b) J-V curve of the flexible Vis-blind NIR OPD in the dark. (c) The photoresponsivity of the flexible Vis-NIR OPD (yellow curve) and Vis-blind NIR OPD (red curve), and the emission spectrum of indoor LED light (blue curve). (d) Photoresponse of the flexible Vis-blind NIR OPD under irradiation at wavelengths of 450, 550, 850, and 950 nm with the same light intensity of 5.0 mW cm^−^^2^. (e) Specific detectivity (D*) of the Vis-NIR (blue curve) and Vis-blind NIR (red curve) OPDs in self-powered mode. (f) Linear dynamic range (LDR) of the flexible Vis-blind NIR OPD under 850 nm illumination and the corresponding responsivities under these light intensities. (g) Phototransient response of the flexible Vis-blind NIR OPD under 850 nm irradiation in self-powered mode. (h) *f_-3dB_* of the Vis-blind NIR OPD under 850 nm LED. (i) The summary of *f_-3dB_* of flexible NIR OPDs.

Building on the high performance of OPDs with PM6:Y6-4Se, we fabricated the flexible OPD element for the Flex-PCI following the established process used for rigid NIR OPDs (Fig. S3b and Methods). To mitigate the visible light interference, here we introduced a finely tuned thin optical adjusting layer (OAL), comprising PCE-10:PC_71_BM blend, on the backside of the flexible NIR OPD to absorb the undesired visible light and obtain the Vis-blind NIR OPD (Fig. 2a and Fig. S11). The detailed fabrication and optimization process of Vis-blind NIR OPDs is presented in the Methods and Text S5. Note that with our strategy and the simple device architecture, it is straightforward and efficient to fabricate narrowband flexible OPDs with customized performance parameters for targeted Flex-PCIs, such as particular spectrum detection range, full-width-half-maximum (FWHM), and even environment-dependent specifications. Such convenient customization can be achieved simply by replacing the active materials and OALs, which has been discussed in our previous study [42].

As the OAL is electrically isolated from the photoactive film, it does not participate in charge dynamic activities in OPDs, allowing the resulting devices to maintain most of the critical performance metrics of original Vis-NIR OPDs, such as low dark current, high responsivity, and fast response speed in the NIR region [42]. As shown in Fig. 2b and c, the representative flexible Vis-blind NIR OPD, with an optimal OAL thickness of 1 µm, shows an ultralow dark current of 0.048 nA cm^−^^2^ and a comparable responsivity (0.34 A W^−^^1^) to the normal flexible Vis-NIR OPD (0.36 A W^−^^1^) at 850 nm in self-powered mode, ensuring low noise current and high sensitivity for faint light detection (Table S7). Moreover, the flexible Vis-blind NIR OPD exhibits a significantly high photocurrent ratio of 186 between NIR light (λ = 850 nm) and visible light (λ = 550 nm) under the same light intensity of 5.0 mW cm^−^^2^ (Fig. 2d), further confirming its robust anti-visible interference capability. Specific detectivity (D*) is another critical figure-of-merit of OPD, quantifying its ability to detect ultraweak optical signals, defined as ${D}^* = \frac{{R\sqrt {AB} }}{{{i}_n}} = \frac{{R\sqrt A }}{{{S}_n}}$, where *A* is 0.2 cm × 0.2 cm, *B* is the bandwidth (1 Hz), *i_n_* is the noise current in A, and *S_n_* is the noise current spectral density in A Hz^−^^1/2^. Owing to its comparable noise current and responsivity to the normal Vis-NIR OPD, the flexible Vis-blind NIR OPD shows a high D* value of over 10^12^ Jones in the NIR region and linear dynamic range (LDR) of over 149 dB at 850 nm, as presented in Fig. 2e, f, and S10b–d. Notably, the flexible Vis-blind NIR OPD maintains a nearly constant responsivity of 0.34 A W^−^^1^ over the light intensity range from 0.43 nW cm^−^^2^ to 12.76 mW cm^−^^2^, making it well-suited for accurate light intensity measurement in practical applications involving finger kinematics and human physiological information detection (Fig. 2f).

The response speed of OPD is a crucial factor for the Flex-PCI, enabling it to precisely capture fast movements, record trajectory and moving speed, and operate naturally, all of which directly influence users’ experience in practical applications. As shown in Fig. 2g, the flexible Vis-blind NIR OPD exhibits a response time of 550 ns under NIR light irradiation (λ = 850 nm), comparable to the normal Vis-NIR OPD and significantly outperforming most reported flexible NIR OPDs (Table S8). The corresponding *f_-3 dB_* is ∼1.32 MHz in the Y6-4Se device (Fig. 2h), which ranks as one of the highest values among the state-of-the-art flexible NIR OPDs (Fig. 2i, Text S4, and Table S8) [43–49]. This ultrafast response speed far exceeds the human response and reaction time (300–600 ms), enabling seamless and natural interaction between humans and machines without delays [17]. Furthermore, the flexible Vis-blind NIR OPD maintained a sustained performance in all critical parameters after bending for 1000 cycles with a curvature radius of 7.5 mm, including noise spectral, responsivity, D*, f_-3dB_, and LDR, showing enormous potential in next-generation flexible electronics (Fig. S12).

### Design and optoelectronic performance of Flex-PCI based on flexible Vis-blind NIR OPDs

**Figure 3.**
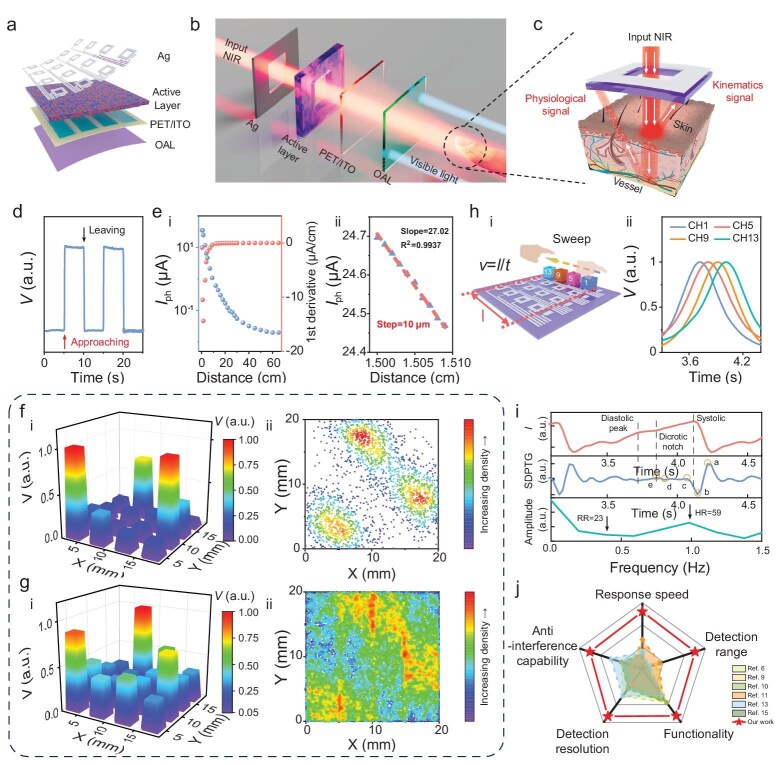
Multifunctional Flex-PCI for finger kinematics and human physiological information detection. (a) Schematic diagram of the Flex-PCI based on flexible Vis-blind NIR OPD. (b) Exploded view of a single Flex-PCI element and its working principle. The LED array (λ = 850 nm) with an emitting area exceeding that of the Flex-PCI was used as a parallel backlight source, with its emission intensity assumed to be uniform to simplify data analysis. (c) Schematic illustration of the Flex-PCI for contactless detection of both finger kinematics and human physiological information. (d) Photoresponse of Flex-PCI when a finger approaches and leaves the device. (e) Photoresponse of the Flex-PCI across a detection range from 0.5 to 60.0 cm using a hand palm and its corresponding first derivative (i); detection range resolution of the device using a hand palm, demonstrating its sensitivity to vertical distance variation. The light intensity of the back panel is 8.3 mW cm^−^^2^ (ii). (f) Photoresponse of the Flex-PCI (i) in the multi-position mode, demonstrating the system's ability to detect multiple positions simultaneously, and the corresponding simulated ray density distribution (ii). (g) Photoresponse of the LP-Flex-PCI (i) in the multi-position mode and the corresponding simulated ray density distribution (ii). (h) Schematic diagram depicting the device's functionality of speed measurement (i); photoresponses of a row of pixels in a Flex-PCI as a stick slides above the device at a distance of 1.0 cm, where the y-axis is the photovoltage of the pixels (ii). (i) Pulse waveform contactlessly measured using the Flex-PCI by fixing a finger over the device at 1 cm; SDPTG analysis of the obtained PPG signal and the corresponding respiratory rate (RR) and heart rate (HR) calculations derived from the FFT analysis of the PPG waveform. (j) The summary and comparison of key metrics of the Flex-PCI with previously reported C-HMIs.

Realizing high-accuracy finger gesture detection, the primary principle is to effectively detect the reflected light from fingers using the Flex-PCI. In addition to the basic highly sensitive OPD elements, it is essential to design sophisticated device structures and system layouts to achieve two primary objectives: (1) to maximize the collection of reflected light for enhanced sensitivity and (2) to minimize the electrical and optical crosstalk effect between neighboring OPD elements. To realize such characteristics, here we developed a prototype Flex-PCI by fabricating a flexible Vis-blind NIR OPD array, featuring square hollow top silver electrodes (0.2 cm × 0.2 cm total size, 0.1 cm × 0.1 cm hollow size), a photoactive layer, a substrate with transparent bottom electrodes (PET-ITO), and an OAL (Fig. 3a). The detailed fabrication process is outlined in the Methods section. A planar LED array (λ = 850 nm) was mounted directly beneath the Flex-PCI and used as a parallel backlight source. The hollow architecture of Flex-PCI allows NIR light from the back panel to vertically pass through the device without attenuation, reflect off the finger's surface, and then reach the OPD elements (Fig. 3b, c, and Text S6). Owing to the strong penetration capability of NIR light, the NIR light penetrating the skin could reach the capillary vessel for potential physiological information detection, such as photoplethysmography (PPG) measurement (Fig. 3b and c).

Moreover, as OPD elements operate independently with negligible electrical crosstalk effect, the system can work in single- and multi-position modes without complex data processing, which will be discussed below. Therefore, detecting the photocurrents of OPD elements makes it possible to obtain finger kinematics and human physiological information simultaneously, such as finger position, height, trajectory, moving speed, heart rate, and respiratory rate. Owing to the simple device architecture and easy solution-processing technique, it is easy to fabricate Flex-PCI with customized pixel sizes, shapes, and resolutions by simply designing the bottom and top electrodes without patterning photoactive materials (Fig. S13 and Text S3). By optimizing the device architecture, photoactive and OAL materials, it is possible to prepare semitransparent Flex-PCIs that integrate seamlessly with state-of-the-art electronic devices for the touchless screen.

To validate the potential applications of our Flex-PCI, we initially measured the photoresponse to the proximity of a palm at varying distances. As shown in Fig. 3d, the device exhibits a rapid and pronounced increase in photocurrent when a palm approaches the device and a sharp decline upon leaving. Remarkably, the device maintains a robust photoresponse across an unprecedented detection range of 0.5–60.0 cm using the palm of a hand, ensuring its potential applications in long-range manipulation (Fig. 3e i and Fig. S14). By fitting the logarithm of photocurrent as a function of distance, a near-linear relationship (${R}^{2\ }$>0.99) was observed across the ranges of 1.0–10.0 cm and 10.0–22.0 cm (Fig. S15). This broad linear response region indicates the device's potential to detect object position with high spatial resolution. The Flex-PCI sensing performance is qualified using sensitivity (*S*) defined as $S = {\mathrm{\Delta }}I/{\mathrm{\Delta }}d$, where ${\mathrm{\Delta }}I$ represents the change in photocurrent, and ${\mathrm{\Delta }}d$ is the displacement of the palm. By conducting the first derivative of the photocurrent vs. distance curve, we obtained the device's sensitivity over a broad detection range from 0.5 to 60.0 cm, peaking at the distance of 1.5 cm (Fig. 3e ⅰ). Within the detection range from 1.505 cm ± 0.005 cm, the relative change of the photocurrent demonstrates a robust linear relationship, with an *R^2^* value of 0.9937, and a high sensitivity of 27.02 μA cm^−^^1^ (Fig. 3e ⅱ). The device achieves a spatial resolution as fine as 10 µm, ranking as the best value among all reported C-HMIs (Table S1). For comparison, we also fabricated a well-established conventional device as a reference device by installing 12 LEDs (λ = 850 nm) at four edges of the same OPD array with the Flex-PCI to produce an LED light plane (LP), named LP-Flex-PCI [16], which shows a narrow detection range of 18.0 cm (Figs S16a and S17b). The theoretical detection ranges, obtained by the optical simulation, for LP-Flex-PCI and Flex-PCI, further corroborate the experimental results and confirm the long-range manipulation capability of our Flex-PCI (Fig. S16b). Detailed simulation procedures are provided in Fig. S16c, d, and Text S7.

### Finger kinematics and human physiological information detection

To further demonstrate the superiority of the position recognition capabilities of our Flex-PCI over the LP-Flex-PCI, we systematically compared their spatial crosstalk effect using a thin metal stick with a similar size to the OPD element. As shown in Fig. S18a and Movie S1, when the metal stick approaches the Flex-PCI, there is a significant photoresponse in the targeted OPD, with negligible responses in neighboring pixels (<30% response of the target OPD element). By conducting a simulation of the light intensity distribution over the device, we found that most of the reflected light is tightly confined within a narrow range in the target OPD, leading to a high spatial resolution and negligible crosstalk photoresponse, which aligns with experimental results (<30% of the light intensity on the target pixel, Fig. S18b–e).

Furthermore, when simultaneously putting three sticks over the Flex-PCI, each stick triggered a distinct photoresponse in its corresponding pixel, with negligible crosstalk effect and signal overlap, significantly demonstrating its multi-position detection capability without requiring a complex algorithm (Fig. 3f i). Such promising results are supported by the optical simulations showing localized light confinements (Fig. 3f ii and S18f–h). Moreover, the light intensities on adjacent OPDs remained below 40% of those on the target OPDs, which is highly consistent with the experimental results (Fig. S18h). In contrast, the conventional LP-Flex-PCI exhibited severe crosstalk in both single- and multi-position modes. As shown in Fig. S17a, the LP-Flex-PCI produced ghost responses in neighboring pixels in the single-position mode (>75% of the light intensity on the target pixel), possibly due to the broad reflected light dispersion over the device. Such a hypothesis is further confirmed by the optical simulation shown in Fig. S17c–e. This issue was exacerbated in the multi-position operation, with severe overlapping signals across adjacent pixels (∼75% of the target responses, Fig. 3g i), which highly aligns with the optical simulation (∼80% of the light intensities on target pixels, Fig. 3g ii, Fig. S17f and g). The superior performance of Flex-PCI stems from its optimized device architecture, which confines incident light within targeted regions. Thus, our design effectively eliminates the need for computationally intensive algorithms to mitigate crosstalk, significantly reducing energy consumption. By simplifying readout circuitry and enhancing signal fidelity, our Flex-PCI establishes a new benchmark for lightweight, flexible photonic systems in contactless human-machine interfaces. Theoretically, the sensitivity and detection range of Flex-PCI could be significantly improved by optimizing the geometry, such as the shape and size of OPD elements and hollow silver electrodes, as well as the parameters of the backlight (position, irradiation direction, and intensity). However, a comprehensive analysis of all these effects on the performance of Flex-PCI is beyond the scope of this work and requires further investigation.

To assess the practical response time of Flex-PCI, we fixed a palm at 1.0 cm over the device to reflect off the modulated NIR irradiation. The response time of the Flex-PCI was 1.6 µs, significantly surpassing all current C-HMIs (Fig. S19a and Table S1). Furthermore, even under modulated light with a frequency of up to 100 kHz, the Flex-PCI shows no attenuation in its photoresponse, demonstrating its strong working capability under high-frequency irradiation (Fig. S19b). Consequently, the Flex-PCI is capable of tracking rapid movement and calculating speed by measuring the displacement of the position (*Δl*) over the moving time (*Δt*) (Fig. 3h i). As shown in Fig. 3h ii, when the stick moves over the Flex-PCI from Channel (CH) 1 to CH13 at a distance of 1.0 cm, the OPD elements are quickly and sequentially triggered. The calculated moving speed of the finger is 41.3 mm s^−^^1^, which aligns well with the experimental setting value (43.0 mm s^−^^1^) with a minor deviation of 4%, ensuring efficient dynamic interaction in real-time applications.

Beyond physical information detection, the Flex-PCI can contactlessly detect vital signals for future VR and AR applications, particularly for information exchange and security. As displayed in Fig. 3i, the Flex-PCI exhibits a reproducible PPG signal when the fingertip is positioned 1 cm above the device, which distinctly shows the systolic peak, dicrotic notch, and diastolic peak. By conducting the Second Derivative Photoplethysmogram (SDPTG), we gain four systolic waves and one diastolic wave for detailed blood information: the a-wave (early systolic positive wave), the b-wave (early systolic negative wave), the c-wave (late systolic reincreasing wave), the d-wave (late systolic redecreasing wave), and the e-wave (early diastolic positive wave). This method serves as a high-resolution, non-invasive tool for assessing arterial stiffness and vascular aging (Fig. 3i) [50,51]. Through fast Fourier transform (FFT) analysis, the time domain of the PPG signals was converted into the frequency domain to derive the heart rate (HR) and respiratory rate (RR). As shown in Fig. 3i, the FFT reveals two peaks at 0.39 and 0.98 Hz, corresponding to an RR of 23 breaths per minute (brpm) and an HR of 58 beats per minute (bpm), respectively. This detailed vital information further proves the high sensitivity of Flex-PCI and its applications in future contactless human-machine interaction and health monitoring. Fig. 3j comprehensively summarizes the critical figures-of-metrics of the state-of-the-art C-HMIs, including detection range, functionality, response speed, detection resolution, and anti-interference capabilities [6,10–13,15], which will be further discussed below, demonstrating that Flex-PCI exhibits significant superiorities over previously reported devices.

### Stability of Flex-PCI under diverse conditions

**Figure 4.**
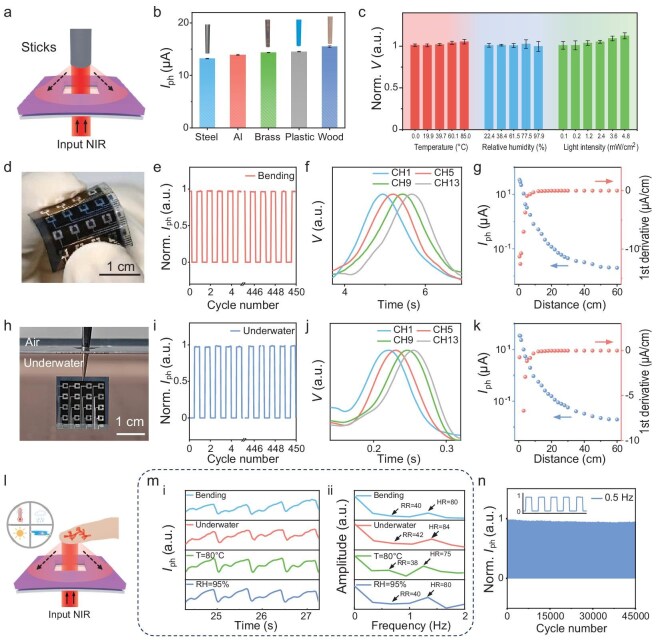
Adaptability and stability of the Flex-PCI under various dynamic ambient and underwater conditions. (a) Schematic diagram of a Flex-PCI manipulation with various materials. (b) Photoresponse of a Flex-PCI when sticks made of steel, aluminum, brass, plastic, and wood with the same diameter of 4.0 mm were positioned at 1.0 cm over the device. (c) Photoresponse of the Flex-PCI under various dynamic ambient conditions, including varied relative humidities, temperatures, and ambient light intensities. (d) Optical image of the Flex-PCI working under a bending condition, and (e) its corresponding photoresponse under 850 nm illumination at a switching frequency of 1 Hz over 15 min. (f) Photoresponse of the Flex-PCI when a finger swipes over the device at a distance of 1 cm in a bending state. (g) Photocurrent of the bent Flex-PCI as a function of the detection range (0.5–55.0 cm), and its corresponding first derivative. (h) Optical image of the Flex-PCI working underwater. (i) Normalized photoresponse of the device under 850 nm illumination with a switching frequency of 1 Hz over 15 min underwater. (j) Photoresponse of the Flex-PCI when a finger swipes over the device at a distance of 1 cm underwater. (k) Photocurrent of the Flex-PCI underwater as a function of the detection distance (0.5–55.0 cm), and its corresponding first derivative. (l) Schematic diagram of contactless physiological information detection. (m) Pulse waveform measured under dynamic ambient conditions (i); the corresponding respiratory rate (RR) and heart rate (HR) derived from FFT analysis (ii). (n) Cycling stability of the Flex-PCI under illumination (λ = 850 nm) at a switching frequency of 0.5 Hz and a light intensity of 240.0 μW cm^−^^2^.

To assess the universal sensing capability of Flex-PCI across various objects, we manipulated the Flex-PCI using five sticks made of distinct materials: steel, aluminum, brass, wood, and plastic, all with a similar shape and diameter of 4 mm (Fig. 4a). The Flex-PCI exhibits a comparable response when fixing these five materials at a distance of 1 cm over the device (Fig. 4b and Movie S1). Notably, there is a minor fluctuation in photoresponse due to the differences in surface properties of these sticks, such as roughness and the material's intrinsic reflective index. These universal sensing characteristics enable users to efficiently interact with Flex-PCI using a wide range of objects beyond mere fingertips. To evaluate the stability and reliability of Flex-PCI in complex and demanding conditions, we subjected the device to a series of rigorous tests. As shown in Fig. 4c, when operating over a temperature range from 0 to 85.0°C, relative humidity between 22.4% and 97.9%, and ambient light irradiation with an intensity up to 4.8 mW cm^−^^2^, our Flex-PCI exhibits exceptional stability and reproducibility with <10% variation of the photoresponse (Text S8). This outstanding stability adequately enables it to operate reliably in various harsh conditions. Even in the bending state with a curvature radius of 7.5 mm, the Flex-PCI maintained a reproducible signal output under the irradiation of an 850 nm LED with a switch frequency of 1 Hz (Fig. 4d and e). When swiping over the bent device using a finger, a fast and sequential response can be observed in Flex-PCI (Fig. 4f). In addition, Flex-PCI under bending state exhibits a strong photoresponse to the palm in close proximity and a sharp decline as the distance increases to 55.0 cm (Fig. 4g and Fig. S20). Moreover, the first derivative of this photocurrent vs. distance curve shows a similar sensitivity with the device in normal condition, indicating good performance retention for the Flex-PCI upon bending.

To realize a stable operation of Flex-PCI underwater, we encapsulated the device by simply using flexible and transparent polyethylene terephthalate (PET) films to prevent water penetration and then immersed it in deionized water (Fig. 4h). As demonstrated in Fig. 4i, the Flex-PCI exhibited a stable and reproducible response to NIR light in underwater conditions. Moreover, the device maintained almost 100% of its initial photoresponse underwater after operating for 450 on/off switching cycles (15 min) of NIR light irradiation (20 mW cm^−^^2^), effectively overcoming the stability issues commonly encountered by other C-HMIs in underwater conditions. Similar to the performance in ambient air, the Flex-PCI shows a fast and sequential response when swiping over the device using a finger in underwater conditions (Fig. 4j). Moreover, the Flex-PCI exhibits a strong photoresponse to the palm in very close proximity and a sharp decline as the distance increases, following the same trend observed in ambient air (Fig. 4k and Fig. S21). Notably, Flex-PCI exhibited a significant response even at a distance of 55.0 cm, ensuring its potential application in long-range underwater manipulation.

Benefitting from high performance and stability, the Flex-PCI consistently exhibits highly stable and reproducible vital signal detection capability, ensuring reliable physiological information monitoring under diverse dynamic conditions (Fig. 4l and m). Furthermore, after operating over 45 000 on/off switching cycles under a light intensity of 240 μW cm^−^^2^ (λ = 850 nm) at a frequency of 0.5 Hz in air, the encapsulated device shows <0.5% attenuation of photoresponse, demonstrating exceptional long-term stability (Fig. 4n). These characteristics collectively ensure the device's practical versatility and reliability in dynamic and demanding conditions, making it a robust solution for diverse real-world scenarios.

### Applications of the high-dimensional security system based on Flex-PC

**Figure 5.**
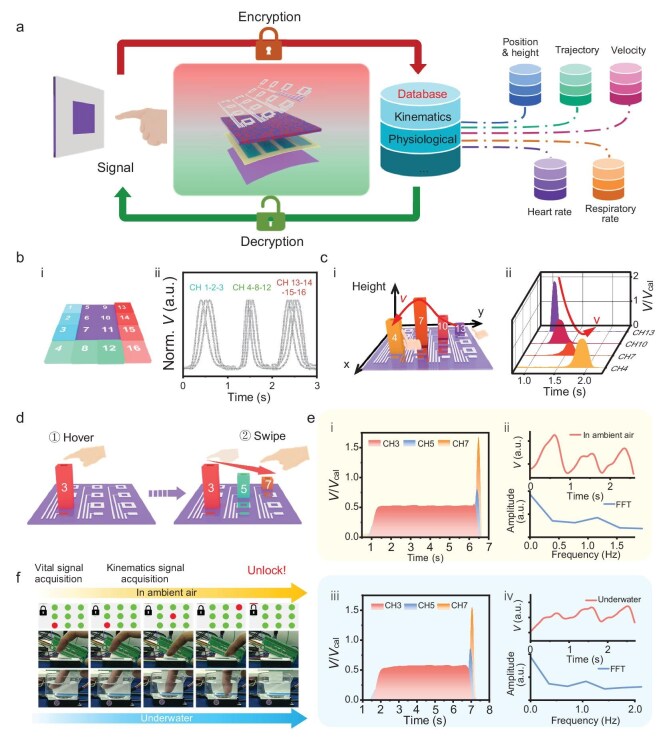
High-dimensional security system based on the Flex-PCI. (a) Schematic diagram of the high-dimensional security system utilizing Flex-PCI. (b) Schematic diagram for the English letter ‘U’ recognized via mapping trigger sequences to sign language (i). Multichannel signal outputs from the Flex-PCI when recognizing the English letter ‘U’ (ii). (c) Schematic diagram of the 4D gesture recognition (i). Multichannel signals from the Flex-PCI when recognizing a swiping gesture with varying heights (ii); the V_cal_ is the calibrated photovoltage measured when the metal stick is swiped over the device at a constant distance of 1 cm (Text S9). (d) Schematic diagram of the high-dimensional authentication process, including kinematics and physiological signal acquisition. (e) The corresponding multichannel signals from the Flex-PCI in ambient air (i) and underwater (iii). Pulse signal obtained from the Flex-PCI with a finger positioned 1 cm over the device and the corresponding FFT results to calculate the respiratory rate (RR) and heart rate (HR) (ii and iv). (f) Demonstration that the system was successfully unlocked via the high-dimensional password in ambient and underwater conditions.

The accurate contactless detection of finger kinematics and human physiological information opens new possibilities for Flex-PCI in the novel contactless security system. Here, we developed a high-dimensional security system based on Flex-PCI, addressing common issues associated with traditional contact-based systems, such as password vulnerabilities, bacterial infection, and bio-information leakage. Figure 5a illustrates the flowchart detailing the encryption and decryption process of the contactless security system. In this system, the finger kinematics and human physiological information, including finger position, height, moving trajectory and speed, heart rate, and respiratory rate, are captured and recorded to create a unique, dynamic, and high-dimensional authentication profile. When a user swipes over the Flex-PCI with accurate pre-set information, the Flex-PCI can precisely and rapidly capture this high-dimensional information and decrypt the password, subsequently granting access. Any deviation from the preset input will trigger a failure in the authentication. To demonstrate the recognition capability of our Flex-PCI, we first used a metal stick to swipe over the system and trace the simple letter ‘U’. Impressively, the element-triggered sequence and corresponding signals were clearly recorded and then mapped to sign language, establishing a direct relationship between the photoresponse of Flex-PCI and sign gestures (Fig. 5b and Movie S2). In addition, the system correctly verified the letters ‘N’ and ‘K’, ensuring its ability to recognize diverse commands (Fig. S22). When a user swipes over the Flex-PCI with a specific finger position, height, trajectory, and speed (Fig. 5c ⅰ and Text S9), the Flex-PCI can accurately record the photocurrent of OPD elements and then translate it to the high-dimensional dynamic gesture information, thereby creating a four-dimensional (4D) password (Fig. 5c ⅱ, Fig. S23, and Movie S3). This dynamic, multi-dimensional authentication approach offers a significant advancement over traditional methods.

To further explore the potential of Flex-PCI in the high-dimensional security system, we designed a system based on Flex-PCI with a resolution of 2.5 PPI (3 × 3 Vis-blind NIR OPD array), minimizing the optical crosstalk effect between neighboring OPD elements (Fig. 5d). A personal security database was first created by recording the user's finger information. Specifically, we fixed the finger at a distance of 2.0 cm over CH3 for a few seconds, followed by swiping over the device in a defined sequence CH3-5-7 with varying finger heights (Fig. 5d, Fig. S24, and Text S9). As shown in Fig. 5e i, the Flex-PCI exhibits a distinct photoresponse and clear pulse waveform, with heart and respiratory rates of 77 bpm and 38 brpm (Fig. 5e ii), respectively. Upon swiping over the Flex-PCI using an accurate gesture, including position, sequence, and height, the system was successfully unlocked (Fig. 5f and Movie S4).

Benefitting from the high stability of Flex-PCI in both ambient and underwater conditions as discussed above, the security system based on Flex-PCI, in principle, should exhibit reproducible and stable performance in these conditions. To further validate this, we immersed the encapsulated Flex-PCI in deionized water and conducted the same security authorization process as in air. As shown in Fig. 5e iii, iv, Fig. S24, and Movie S5, the contactless security system successfully realized the detection of finger kinematics and human physiological information, consistent with that in ambient conditions. The detailed measurement process is presented in Text S9. The slight fluctuation of the pulse waveform baseline can be attributed to the subtle finger displacement during measurement. A long-duration monitoring for human physiological information was conducted both in ambient air and underwater. As depicted in Fig. S25, the pulse waveform remains distinctly visible over 5 min in both environments. The corresponding FFT result demonstrates an RR of 34 brpm and an HR of 66 bpm in ambient air, and an RR of 29 brpm and an HR of 64 bpm underwater, illustrating the Flex-PCI's robust capability for long-period contactless physiological information detection in diverse environments. Thus, this Flex-PCI–based high-dimensional security system does not rely on a single factor but instead integrates a series of dynamic patterns and physiological signals, offering a robust and multi-channel security solution for non-contact application scenarios.

It is worth noting that the various geometry and orientations of fingers or other objects pose challenges for high-accuracy detection in unconstrained environments. To address this, AI-assisted signal processing could be employed to compensate for the full range of geometrical and postural variability encountered in the real world. For example, datasets of responses from a diverse pool of participants—encompassing a wide range of finger sizes, shapes, roughness, and angles relative to the device—can be collected, and a light weight neural network can be trained to map raw data acquired from the Flex-PCI to object shapes and orientations, thereby correcting the geometrical- and postural-dependent reflection effects. These demonstrations highlight the tremendous potential of Flex-PCI for next-generation intelligent electronics, including contactless security systems and other HMI applications.

## CONCLUSIONS

We have developed a prototype Flex-PCI, based on the high-performance Vis-blind NIR OPD array with innovative device architecture, capable of contactless detection for both finger kinematics and human physiological information in self-powered mode. In addition to its exceptional performance and multifunctionalities, the Flex-PCI can also stably and reliably operate across diverse real-world environments, overcoming the longstanding limitations of conventional HMIs. Through systematic explorations of materials, devices, theoretical simulation, and system design, we have established a solid foundation for next-generation C-HMIs. By synergizing finger kinematics with human physiological information, we also designed a high-dimensional security system that offers a secure, intuitive, and hygienic human-machine interaction. Such advanced technology ensures unprecedented security while eliminating the need for physical contact or complex calibration. By integrating high performance, intrinsic flexibility, stability, and multifunctionality, Flex-PCI provides transformative opportunities across AI-driven domains, including security systems, AR/VR interfaces, telehealth platforms, and immersive gaming, which not only advances photonic sensing but also redefines the paradigm for human-machine interaction in an increasingly contactless digital era.

## METHODS

### Fabrication of Flexible Vis-NIR and Vis-blind NIR OPDs

Devices were fabricated on PET substrates with an inverted structure of OAL/PET/ITO/ZnO/PFN-Br/Active Layer/MoO_x_/Ag. First, the ITO-coated PET substrates (17 × 17 mm) were treated under UV exposure for 15 min in a UV-ozone chamber (Jelight Company). To prepare the zinc oxide (ZnO) electron transporting layer, the ZnO nanoparticle solution was spin-coated on the PET-ITO substrates, followed by annealing at 120°C for 10 min in the air, to form a 30 nm electron transporting layer. After that, the PET-ITO substrates with the ZnO layer were transferred to a glovebox filled with nitrogen. A pre-dissolved PFN-Br solution with a concentration of 0.5 mg mL^−^^1^ in methanol was spin-coated onto the ZnO layer. The donor/acceptor mixture solution was then spin-coated on the PFN-Br layer to form a photoactive film with a thickness of ≈150 nm under annealing at 110°C for 5 min. In this case, PM6 and NFAs (1:1.2) were mixed and dissolved in chloroform (the total concentration being 15.4 mg mL^−^^1^ with 0.5% CN). Then, MoO_x_ (3 nm) and Ag (100 nm) films were sequentially deposited on the active layers by thermal evaporation under a vacuum of 2 × 10^−^^5^ Pa. The flexible NIR OPD was finally fabricated, and the effective area of each device was 0.04 mm^2^. As for the flexible Vis-blind NIR OPD, PC_71_BM and PCE-10 (1:1) were mixed and dissolved in chlorobenzene with a total concentration of 40 mg mL^−^^1^ and spin-coated on PET films (the back side of the device) to fabricate OAL to filter the visible light.

### Fabrication of Flex-PCI

The Flex-PCI, based on flexible Vis-blind NIR OPD arrays, was fabricated on patterned PET-ITO substrates (25 mm × 25 mm and 30 mm × 30 mm). The preparation process follows the fabrication procedure of flexible Vis-blind NIR OPDs, with thick top silver electrodes of 300 nm. The evaporation mask consists of 16 central hollowed Ag electrodes with an area of 3 mm^2^ for each (2 mm × 2 mm total size, 1 mm × 1 mm hollow size). Then, the active layer inside the carved Ag electrodes was carefully removed with acetone to ensure that the incident NIR light could pass through the devices. The OAL solution was spin-coated on the back side of the OPD array to absorb visible light.

## Supplementary Material

nwaf303_Supplemental_Files
