# Supplementary material for: Flexible photonic contactless human-machine interface based on visible-blind near-infrared organic photodetectors
Source: Natl Sci Rev. 2025 Jul 26;12(9):nwaf303. doi: 10.1093/nsr/nwaf303 (PMC12416281; doi:10.1093/nsr/nwaf303)
Supplement: nwaf303_Supplemental_Files [file nwaf303_supplemental_files.zip › Supplementary data.pdf]

Supplementary Information

Flexible photonic contactless human-machine interface based on visible-blind near-infrared organic photodetectors

Chen Geng[1, #], Guangkun Song[1, #], Wei Lin[2], Hanzhe Shi[1], Longyu Li[1], Zhaochen Suo[1], Yu Zhu[1], Hao Qin[1], Lin Liu[3], Ruiman Han[4], Yingjun Xia[1], Yanqing Yang[1], Tingting Guo[1], Xiangjian Wan[1], Bo Liu[2], Wangqiao Chen[4], Jing Zhang[1], Ting Zhang[3], Guanghui Li[1, 5, *] and Yongsheng Chen[1, 6, *]

[1]The Centre of Nanoscale Science and Technology and Key Laboratory of Functional Polymer Materials, Institute of Polymer Chemistry, Renewable Energy Conversion and Storage Center (RECAST), College of Chemistry, Nankai University, Tianjin 300071, China;

[2]Institute of Modern Optics, Tianjin Key Laboratory of Micro-scale Optical Information Science and Technology, Nankai University, Tianjin 300350, China;

[3]i-Lab, Suzhou Institute of Nano-Tech and Nano-Bionics (SINANO), Chinese Academy of Sciences, Suzhou 215123, China;

[4]Guangdong Provincial Key Laboratory of Optical Information Materials and Technology, South China Academy of Advanced Optoelectronics, South China Normal University, Guangzhou 510006, China

[5]Academy for Advanced Interdisciplinary Studies, Nankai University, Tianjin 300071, China;

[6]State Key Laboratory of Elemento-Organic Chemistry, Nankai University, Tianjin, 300071, China, Frontiers Science Center for New Organic Matter, College of Chemistry, Nankai University, Tianjin 300071, China

*Corresponding authors. E-mails: ghli1127@nankai.edu.cn; yschen99@nankai.edu.cn

[#]Equally contributed to this work.

CONTENTS

CONTENTS....1
Supplementary Texts....4
Text S1 Materials....4
Text S2 Material Characterizations....4
Text S2.1 Optical Characterization and Analysis....4
Text S2.2 Electrochemical Characterizations and Analysis....5
Text S3 Device Fabrications....5
Text S3.1 Fabrication of Rigid OPDs....5
Text S3.2 Fabrication of Customized Flex-PCI....6
Text S4 OPD Performance Analysis....7
Text S4.1 Key Factors Determining the Noise Current and Response Speed of OPDs....7
Text S4.2 OPD Performance Characterizations....9
Text S4.3 Electronic Characterizations and Analysis of NIR OPDs....9
Text S4.4 Morphology Analysis of OPD....12
Text S5 Performance Optimization of OAL....13
Text S6 Light Source Setup....15
Text S7 Optical Simulation Methodology....15
Text S7.1 Detector Transmittance Mapping....15
Text S7.2 Surface Modeling for Working Distance Simulation....16
Text S7.3 Surface Modeling for Optical Crosstalk Simulation....17
Text S7.4 Light Source Configuration....17
Text S7.5 Light Tracing and Intensity Calculation....17
Text S8 Measurement of Flex-PCI's Adaptability and Stability....18
Text S9 Setup of the High-dimensional Security System....19
Text S9.1 Calibration of the High-dimensional Security System....19
Text S9.2 Measurement of the Contactless Security System....19

Supplementary Figures 21
Figure S1. Chemical structures and the absorption spectrum. 21
Figure S2. Optoelectronic characterizations of OPDs. 22
Figure S3. OPD device structure. 23
Figure S4. Measurement of charge carrier mobilities. 24
Figure S5. Transient photocurrent measurements of devices based on PM6:Y6-4Se and PM6:Y6 analyzed by a one-order exponential decline model. 25
Figure S6. Variation of Voc and Jsc with illumination intensity for the OPDs based on PM6-Y6-4Se and PM6:Y6...26
Figure S7. Measurement of Capacitance Spectra and FTPS-EQE. 27
Figure S8. Measurement of AFM. 28
Figure S9. Measurement of GIWAX. 29
Figure S10. The optoelectronic performance of OPDs based on PM6:Y6-4Se and PM6:Y6...30
Figure S11. Chemical structures and the absorption profile of the OAL materials. 31
Figure S12. The variation of the Vis-blind NIR OPD's performance parameters after a thousand bending cycles. 32
Figure S13. Large-area fabrication of Flex-PCIs. 33
Figure S14. Raw data for measuring the working distance of Flex-PCI in ambient air...34
Figure S15. Linear fitting of the logarithm of photocurrent as a function of distance over the ranges of 1.0–10.0 cm and 10.0–22.0 cm. 35
Figure S16. The detection range of LP-Flex-PCI and the optical simulation of the working range. 36
Figure S17. The optical crosstalk simulation of LP-Flex-PCI. 37
Figure S18. The optical crosstalk simulation of Flex-PCI. 38
Figure S19. Phototransient performance of the Flex-PCI. 39
Figure S20. Raw data for measuring the working distance of Flex-PCI under the

bending state. 40
Figure S21. Raw data for measuring the underwater working distance of Flex-PCI. 41
Figure S22. Gesture recognition of Flex-PCI. 42
Figure S23. V_{cal} for 4D password system. 43
Figure S24. V_{cal} in ambient air and underwater. 44
Figure S25. The long-time period pulse waveform measurement. 45
Figure S26. Nyquist plots of devices based on PM6:Y6-4Se and PM6:Y6. Inset: The equivalent-circuit model employed for fitting of EIS data. 46
Supplementary Tables 47
Table S1. Performance summary of literature-reported C-HMIs. 47
Table S2. Summary of energy levels and spectral absorption properties of donors and acceptors. 48
Table S3. The parameters of the space-charge limited current (SCLC). 49
Table S4. The parameters of OPDs calculated via Mott-Shockley analysis. 50
Table S5. Summary of E_u for OPDs based on PM6:Y6-4Se and PM6:Y6. 51
Table S6. The detailed information from GIWAXS. 52
Table S7. The figure of merits of rigid and flexible OPDs. 53
Table S8. Performance summary of literature-reported flexible NIR OPDs. 54
References 55

Supplementary Texts

Text S1 Materials

Y6-4Se was synthesized according to the previous report [1]. Other chemicals used in this work were directly purchased and used without further purification. Polymer donor PM6 was from Solarmer Material (Beijing) Inc.. PFN-Br was purchased from Luminescence Technology Corp.. PCE-10 and $PC_{71}BM$ for OAL were from 1-Material Inc. and American Dye Source, Inc., respectively. Chloronaphthalene and MoO_x were purchased from Sigma Aldrich. ITO-coated glass and PET were from Advanced Election Technology Co., Ltd. Chlorobenzene was supplied by Aladdin (China). 2-methoxyethanol was obtained from J&K Scientific Ltd.. Zinc acetate and ethanolamine were from Thermo Fisher Scientific Inc.

Text S2 Material Characterizations

Incorporating highly polarizable selenium (Se) atoms onto the backbone of organic conjugated materials has been proven to be an effective way to decrease their band gap [1,2]. The better electron cloud delocalization of Se causes larger orbital overlap in π-conjugated systems, resulting in enhanced quinoidal character [3]. Moreover, replacing S with Se could keep the skeleton characteristics of molecules to the maximum and generate minimal impacts on molecular geometries and further intermolecular packing networks [1].

Text S2.1 Optical Characterization and Analysis

The UV-visible (UV-vis) spectra of chloroform solution and films were tested on an Agilent Technologies Cary 5000 spectrophotometer. Each film sample was spin-coated on quartz substrates from its chloroform solution.

The maximum absorption wavelength of Y6-4Se is 766 nm in solution,

corresponding to a 35 nm redshift compared with Y6. In thin films, an obvious redshift of ~ 85 nm can be observed for Y6 and Y6-4Se neat films with absorption peaks located at 817 nm and 850 nm, respectively, suggesting strong intermolecular interactions (**Figure S1b**).

Text S2.2 Electrochemical Characterizations and Analysis

The measurement of the cyclic voltammeter (CV) was performed on an LK98B Ⅱ Microcomputer-based Electrochemical Analyzer using a glossy carbon electrode as the working electrode, a saturated calomel electrode as the reference electrode, and a Pt wire as the counter electrode. 0.1 mol L^{-1} tetrabutylammonium hexafluorophosphate (Bu_4NPF_6) in a solution of acetonitrile was used as the supporting electrolyte.

The PM6, Y6, and Y6-4Se films in the electrolyte were scanned at a rate of 100 mV s^{-1}. The potentials were calibrated with the ferrocene/ferrocenium redox couple (Fc/Fc^+) at 4.8 eV with respect to the vacuum level. The highest occupied molecular orbital (HOMO) and the lowest unoccupied molecular orbital (LUMO) energy levels were calculated using the following equations:

$$E_{HOMO} = -\left(E_{ox} + 4.8 - E_{Fc/Fc^+}\right)\, eV \# (S1)$$

$$E_{LUMO} = -\left(E_{red} + 4.8 - E_{Fc/Fc^+}\right)\, eV \# (S2)$$

Cyclic voltammeter (CV) measurements of PM6, Y6, and Y6-4Se are shown in **Figures S2a-c**. As shown in **Figure S2d** and **Table S2**, the HOMO/LUMO energy levels of PM6, Y6 and Y6-4Se are -5.58/-3.74 eV, -5.69/-3.87 eV and -5.62/-3.86 eV, respectively. The reduced bandgap of Y6-4Se is mainly due to its up-shifted HOMO.

Text S3 Device Fabrications

Text S3.1 Fabrication of Rigid OPDs

Devices were fabricated on ITO/glass substrates with an inverted structure of

ITO/ZnO/PFN-Br/Active Layer/MoO_x/Ag. Firstly, the ITO-coated glass substrates (17 mm × 17 mm) were sequentially pre-cleaned in an ultrasonic bath with detergent water, deionized water, acetone, and isopropyl alcohol for 20 min each, then dried via N_2 gas. Subsequently, ITO/Glass substrates were treated under UV exposure for 15 min in a UV-ozone chamber (Jelight Company). To prepare the sol-gel zinc oxide (ZnO) electron transporting layer, 0.05 g of zinc acetate was dissolved in 2 mL of 2-methoxyethanol with 14 μL of ethanolamine and stirred at room temperature for 12 h. The ZnO precursor solution was spin-coated on the ITO substrates, followed by annealing at 200 ℃ for 50 min in the air. After that, the ITO substrates with the ZnO layer were transferred to a glovebox filled with nitrogen. A thin film of PFN-Br was formed by spin-coating a 0.5 mg mL^{-1} PFN-Br solution in methanol onto the ZnO layer. Then the donor/acceptor mixture solution (the ratio of donor to acceptor is 1:1.2 and the total concentration is 13.2 mg mL^{-1} in chloroform with 0.5% CN) was spin-coated on the PFN-Br layer to form a photoactive film with a thickness of around 130 nm followed by annealing at 110 ℃ for 5 min. Finally, MoO_x (3 nm) and Ag (100 nm) films were sequentially deposited on the blend films by thermal evaporation under a vacuum of 2×10^{-5} Pa. The effective area of each device was 0.04 mm^2.

Text S3.2 Fabrication of Customized Flex-PCI

Devices were fabricated on PET substrates with an inverted structure of OAL/PET/ITO/ZnO/PFN-Br/Active Layer/MoO_x/Ag. Firstly, the patterned ITO-coated PET substrates with different sizes (38.1 × 38.1, 76.2 × 76.2 mm) were treated under UV exposure for 15 min in a UV-ozone chamber. To prepare the zinc oxide (ZnO) electron transporting layer, the ZnO nanoparticle solution with a concentration of 15 mg mL^{-1} was blade-coated on the PET-ITO substrates, followed by annealing at 120 ℃ for 10 min in the air. The gap between the substrate and the blade is 200 μm, the coating velocity is 20 mm s^{-1}, and the substrate temperature is about 60 ℃. After that, a pre-dissolved PFN-Br solution with a concentration of 0.5 mg mL^{-1} in methanol was blade-coated onto the ZnO layer to modify the interfacial

properties. Then, the active layer solution (PM6:Y6-4Se = 1:1.2) with a total concentration of 19.8 mg mL^{-1} in chlorobenzene was blade-coated onto PET/ITO/ZnO/PFN-Br surface in the air with the gap of 200 μm and coating velocity of 15 mm s^{-1} when the substrate temperature is about 70 ℃, followed by annealing at 110 ℃ for 5 min. Then, MoO_x (3 nm) and Ag (300 nm) films were sequentially deposited on the active layers by thermal evaporation under a vacuum of 2×10^{-5} Pa.

The evaporation mask of Ag consists of various sizes and shapes of central hollowed Ag electrodes to fabricate Flex-PCIs with different resolutions (3.33 ppi and 4.20 ppi) and element shapes (square and circle). Then, the active layer inside the carved Ag electrodes was carefully removed with acetone to ensure the incident NIR light could pass through the devices. $PC_{71}BM$ and PCE-10 (1:1) were mixed and dissolved in chlorobenzene with a total concentration of 40 mg mL^{-1} and blade-coated on PET films (the back of the device) to fabricate OAL.

Text S4 OPD Performance Analysis

Text S4.1 Key Factors Determining the Noise Current and Response Speed of OPDs

1. To meet the critical demands for the optical and electrical characteristics of Flex-PCI, it is imperative to optimize photoactive materials for a high-sensitivity and high-speed NIR OPD. Previous studies have shown that thermally excited charges, which are primarily correlated to effective bandgap (E_{eff}) [4,5], trap states [6], and energetic disorder [4,7] in organic materials, dominate the noise current in OPDs operating in photovoltaic mode. In BHJ of OPDs that consists of donor and acceptor materials, the effective bandgap (E_{eff}) is determined by the difference between HOMO of the donor and LUMO of the acceptor. Besides, organic semiconductors exhibit a broad energy state distribution extending into the bandgap, resulting in the formation of bandtail states occupied by a relatively low concentration of thermally excited carriers that are spatially far from the band

edges. As a result, the effective barriers to thermally generated charges decrease as the energy state distribution increases, leading to rising free charges and noise current [4].

2. Another factor contributing to the dark noise current is the presence of traps in the BHJ blend, which are involved in charge generation and recombination [6]. According to the Shockley-Read-Hall (SRH) theory [8], an electron is thermally excited from the donor HOMO (CT ground state) to a trap state (r_1) located in the middle of the bandgap. This is followed by a second excitation and further release to the acceptor LUMO (r_2), contributing to the concentration of thermally excited charges [9,10]. In this thermal charge generation and recombination process, the trap center occupied by a hole (or electron) may recombine with an electron (or hole) from the acceptor LUMO (or donor HOMO) or emit to the donor HOMO (or acceptor LUMO) until equilibrium is reached by equal capture and emission rates for holes and electrons [8]. Accordingly, the thermal charge generation increases linearly with the concentration of trap states, therefore, a higher concentration of trap states generally results in a higher dark noise current [11]. Hence, reducing energetic disorder and trap states can effectively suppress the generation of thermally excited charges in the organic film, thereby reducing the noise current.

3. Additionally, charge transport in highly disordered amorphous organic films generally occurs via the hopping process, requiring sufficient energy to overcome the barriers caused by energetic disorder, as described by the Arrhenius-like law [12]:

$$\mu_0 = \mu_\infty \exp\left(-\frac{\Delta}{k_B T}\right) \# \#(S3)$$

Here, Δ represents the activation energy, which increases with the amount of disorder. Assuming that the traps are homogeneously dispersed, the mobility can be expressed as

$$\mu = \mu_0 \alpha \exp\left(-\frac{E_t}{kT}\right) \# \#(S4)$$

where E_t is the trapping energy and α is the ratio of the density of delocalized

levels available for transport to the density of traps.

Therefore, reducing trap states and energetic disorder in photoactive films can effectively suppress the noise current and enhance charge mobilities, thus leading to high sensitivity and response speed in OPDs.

Text S4.2 OPD Performance Characterizations

Rigid OPDs were tested in an electromagnetic shielding box to decrease the external electromagnetic interference. The semiconductor device analyzer (KEYSIGHT, B1500A) was used to record dark current and photocurrent. The responsivity of OPD was carried out using a QE-R Solar Cell Spectral Response Measurement System (Enli Technology Co. Ltd.). The noise current was measured by the semiconductor device analyzer and processed by FFT analysis. The transient response was recorded by a digital oscilloscope (Tektronix MDO32), and the pulse light was modulated by a function generator (Rigol, DG 1022). The multi-channel signals of OPD arrays were recorded by a data acquisition (DAQ) device (USB-6211, National Instruments).

Text S4.3 Electronic Characterizations and Analysis of NIR OPDs

1. Energetic disorder analysis: To investigate the factors contributing to the high sensitivity and high response speed of Y6-4Se-based NIR OPD, a comprehensive analysis of the electronic performance of NIR OPDs was conducted. Urbach Energy (*E_u*), as an important parameter to characterize the energetic disorder [13], was quantificationally investigated in the OPDs with PM6:Y6-4Se and PM6:Y6 blends, respectively, using Fourier transform photocurrent spectroscopy-external quantum efficiency (FTPS-EQE). The calculation formula of E_u is:

$$E_u(E) = \left[\frac{dln(EQE)}{dE}\right]^{-1} \# \#(S5)$$

where E is the photon energy and EQE is the external quantum efficiency. The FTPS-EQE was carried out on an Enlitech FTPS PECT-600 instrument. The PM6:Y6- and PM6:Y6-4Se-based devices were used for FTPS-EQE measurement

directly. As shown in **Figures S7a**, **b** and **Table S5**, the PM6:Y6-4Se-based OPD shows lower E_u (17.09 meV) than that of OPD based on PM6:Y6 (18.01 meV), indicating a reduction in energetic disorder and less extension of the band-tail states into the bandgap. To our knowledge, the value of 17.09 meV is among the lowest E_u reported for organic semiconductor materials. The lower E_u of PM6:Y6-4Se can be attributed to the higher molecular rigidity and stronger intermolecular interaction of Y6-4Se.

2. Trap states analysis: Moreover, to investigate the trap states in the optimized devices, capacitance-voltage (C-V) measurements were conducted. An impedance analyzer (Zennium E41081) was used to obtain capacitance-voltage (*C-V*) characteristics in dark conditions under room temperature. The applied AC voltage and frequency were set as 100 mV and 10 kHz, respectively. According to the Mott-Shockley analysis, the trap density (N_A) and the space charge width (W) were calculated using the following equations [14]:

$$N_A = \frac{-2}{q\varepsilon_r\varepsilon_0 A^2}\left(\frac{dV}{dC^{-2}}\right) \#(S6)$$

$$W = \sqrt{\frac{2\varepsilon_r\varepsilon_0(V_{bi} - V)}{qN_A}} \#(S7)$$

where q is the charge of an electron, ε_r is the relative dielectric constant of the active layer, $\varepsilon_0$is the vacuum permittivity, A is the device area and V_{bi} is the built-in voltage. As shown in **Figures S7c**, **d** and **Table S4**, the OPD based on PM6:Y6-4Se shows a lower trap density of 4.04×10^{15} cm^{-3} than OPD with PM6:Y6 blend (4.14×10^{15} cm^{-3}).

3. The charge mobilities measurement and analysis: The hole and electron mobilities of the devices were evaluated using the space charge limited current (SCLC) method. Hole mobilities were measured with a device structure of Glass /ITO/PEDOT:PSS/Active layer/MoO_x/Ag and electron mobilities were measured with a device structure of ITO/ZnO/Active layer/PDINO/Ag. The corresponding charge mobilities were estimated from a fit to the Mott-Gurney square law:

$$J = \frac{9\varepsilon_0\varepsilon_r\mu V^2}{8d^3} \#(S8)$$

where J is the current density, ε_r is the relative dielectric constant of the active layer, ε_0 is the vacuum permittivity, d is the thickness of the active layer, and μ is the mobility of the hole or electron. Benefitting from the less trap states and energetic disorder, the NIR OPD based on PM6:Y6-4Se shows higher hole/electron charge mobilities of 5.49×10^{-4} cm^2 V^{-1} s^{-1} and 6.01×10^{-4} cm^2 V^{-1} s^{-1} than the values of PM6:Y6 films (5.10×10^{-4} cm^2 V^{-1} s^{-1}/5.44×10^{-4} cm^2 V^{-1} s^{-1}), calculating via a space-charge-limited current method (**Figure S4** and **Table S3**). The higher charge mobilities in Y6-4Se-based OPDs can improve the device response speed.

4. Charge recombination analysis: The short-circuit current density (J_{sc}) follows the power law $J_{sc} \propto P_{light}^{\alpha}$, where an exponent α approaching unity signifying minimal bimolecular recombination [15]. As shown in **Figure S6**, the OPD based on PM6:Y6-4Se exhibits an α of 99.48%, higher than the value of the device based on PM6:Y6 (99.03%), indicating its reduced bimolecular recombination. Moreover, the relationship between open-circuit voltage (V_{oc}) and P_{light} can be expressed as $V_{oc} \propto \frac{nkT}{q}\ln(P_{light})$, where k is the Boltzmann constant, T is the absolute temperature, and q is the elementary charge [16,17]. In **Figure S6**, the PM6:Y6-4Se-based OPD shows a slope of $1.22\,kT/q$, lower than that of the PM6:Y6-based device ($1.30\,kT/q$), demonstrating effective suppression of trap-assisted recombination.
5. RC time constant analysis: Besides mobility, there are some additional factors contributing to response speed, such as series resistance. The frequency response is normally affected by the resistor-capacitor (RC) time constant of the circuit and carrier transit time [18-20]. The real -3 dB bandwidth ($f_{-3\,dB}$) is a competition of RC time and charge transport, which can be calculated as

$$\frac{1}{f_{-3\,dB}^2} = \frac{1}{f_t^2} + \frac{1}{f_{RC}^2} \#(S9)$$

where f_t is the carrier transit time limited bandwidth, and f_{RC} is the RC limited

bandwidth. The f_t is given by

$$f_t = \frac{3.5}{2\pi t_r} \#(S10)$$

where t_r is the carrier transit time, relevant to the mobility [21]. The f_{RC} can be calculated as

$$f_{RC} = \frac{1}{2\pi RC} \#(S11)$$

where R is the total series resistance, including the OPD's series resistance (R_s) and the load resistance of the circuit, and C is the junction capacitance of the device. The value of R_s was extracted through electrochemical impedance spectroscopy (EIS) as shown in **Figure S26**. The series resistances and capacities of OPDs based on PM6:Y6-4Se and PM6:Y6 derived from EIS are 24 and 33 Ω, and 2.31 and 2.96 nF, respectively, contributing to the high response speed and wide $f_{-3\ dB}$ for the device based on PM6:Y6-4Se.

6. Carrier extraction time analysis: As shown in **Figure S5**, we conducted transient photocurrent (TPC) to evaluate the carrier extraction time of the OPDs [20,22]. The OPD with PM6:Y6-4Se blends exhibits a shorter carrier extraction time (0.40 μs) than that based on PM6:Y6 (0.63 μs), demonstrating the superior charge transport properties of PM6:Y6-4Se.

Text S4.4 Morphology Analysis of OPD

To further investigate the reasons for the reduced trap states and energetic disorder and enhanced charge mobility in OPD with PM6:Y6-4Se blend, atomic force microscopy (AFM) and grazing-incidence wide-angle X-ray scattering (GIWAXS) were conducted to study the morphologies of blend films.

1. AFM analysis: AFM images were obtained from a Bruker Dimension Icon atomic force microscope in tapping mode. All the film samples were spin-coated on Glass/ITO/ZnO/PFN-Br substrates under the same conditions as NIR OPD fabrication. As shown in **Figure S8,** the blend film of PM6:Y6-4Se shows a distinct fibrillar network interpenetrating structure, which is expected to decrease

charge recombination and promote charge separation and transport. The blend film based on PM6:Y6-4Se exhibits a larger root-mean-square (RMS) roughness of PM6:Y6-4Se (3.14 nm) than PM6:Y6 (0.71 nm), which should result from the strong crystalline property of Y6-4Se.

2. GIWAX analysis: GIWAXS measurement was carried out at Xeuss 3.0 SAXS/WAXS. Each sample was prepared on Si/ZnO/PFN-Br substrate and irradiated at a fixed incident X-ray angle of 0.2 ° with a dwell time of 1800 s. From the GIWAXS results of the blend films (**Figure S9** and **Table S6**), the film of PM6:Y6-4Se shows a stronger π-π stacking (010) peak in the out-of-plane (OOP) direction at about 1.68 $Å^{-1}$ than that of the PM6:Y6 blend (1.65 $Å^{-1}$), with a smaller π-π stacking distance (*d*, 3.74 Å) than that of the PM6:Y6 blend (3.81 Å), indicating an enhanced molecular packing in the PM6:Y6-4Se blend film. According to the Scherrer equation, the crystal coherence lengths (CCL) of the PM6:Y6-4Se blends reach 29.49 Å in the OOP direction, which is larger than that of PM6:Y6 film (27.05 Å). To sum up, the PM6:Y6-4Se film shows reduced π-π stacking distance and enhanced CCL, indicating an appropriate phase morphology with reduced disorder and enhanced crystallinity.

Consequently, the introduction of Se in the core unit of Y6 acceptor efficiently optimizes the film morphology and enhances intermolecular stacking due to the strong interaction of Se atoms, leading to reduced trap states and energetic disorder and enhanced charge mobility in PM6:Y6-4Se film, simultaneously. As a result, the OPD based on PM6:Y6-4Se shows notably faster response speed and higher sensitivity than that based on PM6:Y6.

Text S5 Performance Optimization of OAL

As shown in **Figure 2c**, the visible indoor light spectrum spans from about 400 nm to 750 nm, in which Vis-NIR OPD based on PM6:Y6-4Se blend exhibits an obvious response. To block the interference from visible light, we chose $PC_{71}BM$ and PCE-10

blend as OAL. Considering that the neat $PC_{71}BM$ and PCE-10 thin films show strong and complementary absorption ranging from 400 to 600 nm and from 600 to 750 nm, respectively, we choose their ratio as 1:1, and the resulting blend film shows an absorption profile fully covering the visible light spectrum (**Figure S11c**).

According to the Beer-Lambert Law, the intensity of light passing through a film is directly associated with its molar extinction coefficient and light passing length, which is described as follows:

$$I(\lambda) = I_0(\lambda) \times 10^{-\varepsilon(\lambda)l} \# (S12)$$

where $I(\lambda)$ is the light intensity passing through the film, I_0 refers to the input light intensity, $\varepsilon(\lambda)$ represents the molar extinction coefficient, and l denotes the light path, *i.e.*, film thickness, here. OALs with different thicknesses were fabricated by tuning the concentration of $PC_{71}BM$: PCE-10 solution and speed during the spin-coated process. The thickness of OAL was measured using a Veeco Dektak 150 profilometer. As shown in **Figure S11d**, these films exhibit an almost constant and high molar extinction coefficient of about 2.6×10^4 cm^{-1}. The responsivities of devices with different OAL thicknesses were measured and are shown in **Figure S11e**. As the thickness increases, the response of OPD to visible light decreases significantly. And when the thickness reaches 1000 nm, the Vis-blind NIR OPD exhibits a negligible response in the visible range (1.66×10^{-4} A W^{-1} @450 nm) and maintains a comparable responsivity at 850 nm (0.41 A W^{-1}) to the normal Vis-NIR OPD (0.45 A W^{-1}). The total concentration of OAL solution was finally determined to be 40 mg mL^{-1} in chlorobenzene to maintain a balance between visible-light blocking capability and solution-processability. The fine-tuned OAL with a thickness of ~ 1000 nm shows a high absorbance of 2.44, which means 99.6% of the visible light can be absorbed. As a result, the Vis-blind NIR OPD with such an optimized OAL presents a highly visible-blind response.

Text S6 Light Source Setup

For Flex-PCI, an 850 nm planar LED array was mounted beneath as a parallel backlight source, and its emission intensity is assumed to be uniform to simplify data analysis. The emitted collimated NIR light could only pass vertically through the Flex-PCI, reflect off the user's finger surface, and then reach the OPDs in Flex-PCI. The area of the planar LED array is larger than that of the Flex-PCI. For LP-Flex-PCI, we installed 12 LEDs (λ = 850 nm) along the four edges of the same OPD array used in the Flex-PCI, generating a light plane parallel to the OPD array (**Figure S17b**).

Text S7 Optical Simulation Methodology

A theoretical simulation was conducted to further demonstrate our device's advantages over those based on LP-Flex-PCI structure, including a longer working distance and reduced optical crosstalk, using the commercial software MATLAB R2023b. Two models were constructed, one with LEDs positioned beneath and the other with LEDs positioned around the Flex-PCI.

Text S7.1 Detector Transmittance Mapping

In this study, a spatial resolution of 0.1 mm was selected to balance computational accuracy and efficiency. The transmittance of each layer was defined individually based on the actual device structure. The transmittances of the interface layers, including ZnO nanoparticles (ZnO NPs) as the electron transport layer (ETL) and MoO_x as the hole transport layer (HTL), were assumed to be 1. For 850 nm near-infrared (NIR) light, the transmittance values of the other layers were set as follows:

- OAL: 0.97
- PET: 0.98
- ITO: 0.99

• Active layer: 0.6

Notably, the transmittance of the hollow regions in both the active layer and silver electrode was set to 1. The total transmittance of the Flex-PCI was calculated as the product of the transmittances of all individual layers.

Subsequently, the transmittance distribution was configured to reflect the actual device structure. A nested loop approach was employed to generate a 4×4 periodic array, simulating the patterned distribution of the active layer. Through mask subtraction, normalization, threshold filtering, and further use of nested loops, a 4×4 periodic array with centrally hollowed regions was created, representing the patterned distribution of the silver electrodes, whose transmittance was set to 0 (**Figure S16c**).

Text S7.2 Surface Modeling for Working Distance Simulation

To accurately model the human palm surface, the reflective area is first defined as an 80×80 mm plane, on which periodic arcs are employed to replicate the texture of the palm. The radius of these arcs is set to be 300 mm, with a period of 0.5 mm and a duty cycle of 0.8. For each period along the Y-axis, an arc is generated to represent the ridge positions. The radius of each arc is randomly perturbed to approach the real state of the texture. Building upon these ridges, valleys are introduced with an offset determined by the product of the period and duty cycle, further contributing to a more natural texture.

Gaussian-distributed roughness is applied to the texture to simulate the micro-roughness of the palm surface. The mean value of the roughness is set to be 0 mm to avoid any overall shift in surface height, while the standard deviation is specified as 0.01 mm to simulate the surface details characteristic of the palm.

Finally, the texture and roughness are combined to generate a refined model that faithfully represents the palm’s surface (**Figure S16d**).

Text S7.3 Surface Modeling for Optical Crosstalk Simulation

In accordance with the experimental design, a single metal rod or an array of three metal rods, each with a diameter of 4 mm, was positioned 10 mm above the Flex-PCI to simulate single and multi-position operations. For the single-position operation, the center of the circular metal rod plane was located at (-6.5, -6.8, 10) (**Figures S17c** and **18b**). For the multi-touch operation, the centers of the three circular planes were positioned at (-1.95, -6.8, 10), (7.1, 1.2, 10), and (-6.5, 5.25, 10), respectively (**Figures S17f** and **18f**). The roughness of these circular planes was consistent with the settings used in the aforementioned **Surface Modeling for Working Distance Simulation** section.

Text S7.4 Light Source Configuration

In our configuration, light is emitted from beneath the Flex-PCI, passes through the device, and is then reflected off the object. As a result, the positions and angles of the light source do not need to be explicitly defined. Instead, light reflections are indirectly modeled using a surface reflection model, where the roughness and texture of the reflecting surface influence the paths of the light. For the LP-Flex-PCI configuration, the properties of the LED sources are explicitly defined. According to the experimental setup, twelve near-infrared LEDs are positioned around the Flex-PCI, 1 mm above the device. Each LED has a beam divergence of 120 °. The coordinates of the twelve LEDs are as follows: (-10, -5, 1), (-10, 0, 1), (-10, 5, 1), (10, -5, 1), (10, 0, 1), (10, 5, 1), (-5, -10, 1), (0, -10, 1), (5, -10, 1), (-5, 10, 1), (0, 10, 1), and (5, 10, 1) (**Figure S17b**).

Text S7.5 Light Tracing and Intensity Calculation

The light tracing process primarily involves several steps. First, the surface normal components in the x and y directions are calculated by computing the height differences of the adjacent target surface along the x and y axes. Subsequently, using

these normal components in conjunction with the original coordinates x and y, the coordinates of the reflected light impact points can be calculated. Through logical filtering, the valid rays located within the region of the Flex-PCI are retained, and those in areas with 0 transmittance, i.e., the silver electrode regions, are removed. Finally, the distribution of reflected rays is visualized using a scatter plot, with the transmittance values mapped as colors to represent the intensity of the rays. Additionally, the total light intensity within an Ag electrode region can be extracted. A function relating the light intensity to the distance between the reflecting object and the Flex-PCI is further established.

Text S8 Measurement of Flex-PCI's Adaptability and Stability

Five thin sticks with similar shapes and sizes but different materials, including steel, aluminum, brass, wood and plastic, were used to test the universality of Flex-PCI. They were placed 1 cm above the Flex-PCI, and multichannel signals of the device were measured.

When testing the adaptability and stability of Flex-PCI under various relative humidities and temperatures, the device was put in a sealed acrylic box. The real-time relative humidity and temperature were monitored by a hygrothermograph with its probe placed near the device. Water was sprayed with an airbrush to control the humidity. In addition, we used an electric heater to increase the temperature. As for the low-temperature environment, the device was put in a refrigerator.

Before we immersed the Flex-PCI in deionized water, the device was encapsulated by sticking PET films on both sides of the device to prevent the leak of water.

The flexible devices were manually bent for 1000 cycles using a specific bending mold with a curvature radius of 7.5 mm in a glovebox filled with N_2. The optoelectronic performances of flexible OPDs, including noise spectral density, responsivity, specific detectivity, -3 dB bandwidth, and LDR, were tested before and

after bending tests.

Text S9 Setup of the High-dimensional Security System

Text S9.1 Calibration of the High-dimensional Security System

The test begins by sequentially sliding the finger over the device in the order of CH 13-10-7-4 at a constant height (~ 1 cm) to obtain the calibration photoresponse of each OPD (V_{cal}), which is then stored as a standard reference in the database. To streamline the authentication process, we categorized the finger height into three levels: the high level, where the finger height is greater than 1 cm, the medium level, where the finger height is about 1 cm, and the low level, where the height is less than 1 cm. We can approximate the finger height by measuring the photoresponses of the OPD elements (V) and calculating the ratio of V/V_{cal} (**Figures S23** and **S24**).

Text S9.2 Measurement of the Contactless Security System

To create a personal database, we first calibrate the photoresponse of Flex-PCI following the procedure detailed in **Calibration of the High-dimensional Security System** section. Then we fix the finger over the targeted OPD (e.g., CH 3) at a distance of ~ 2 cm for 5 seconds to collect PPG data and derive the heart rate and respiratory rate through FFT analysis. Next, the finger is swiped over the Flex-PCI following a pre-set gesture (e.g., CH 3-5-7) at varying heights. By measuring the photoresponses of the OPD elements and comparing them with the V_{cal} of corresponding pixels, we can capture gesture-related information such as finger position, height, trajectory, and moving speed.

In the demonstration shown in **Figure 5d**, we first hover the finger over CH 3 to collect the PPG signal, followed by a swipe across the Flex-PCI along the trace of CH 3-5-7, with the finger height gradually decreasing. As shown in **Figure 5e**, the V/V_{cal} ratio increases gradually from 0.5 to 1.5, indicating a decreasing height of the finger

trajectory, which is consistent with the pre-set gesture information.

Supplementary Figures

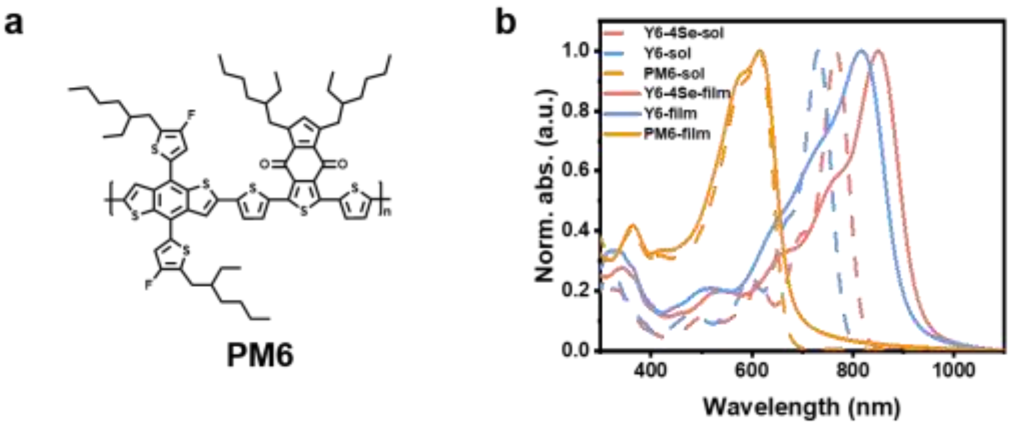

Figure S1. Chemical structures and the absorption spectrum. (a) The chemical structures of PM6. (b) The normalized solution and thin film absorption profiles of PM6, Y6, and Y6-4Se.

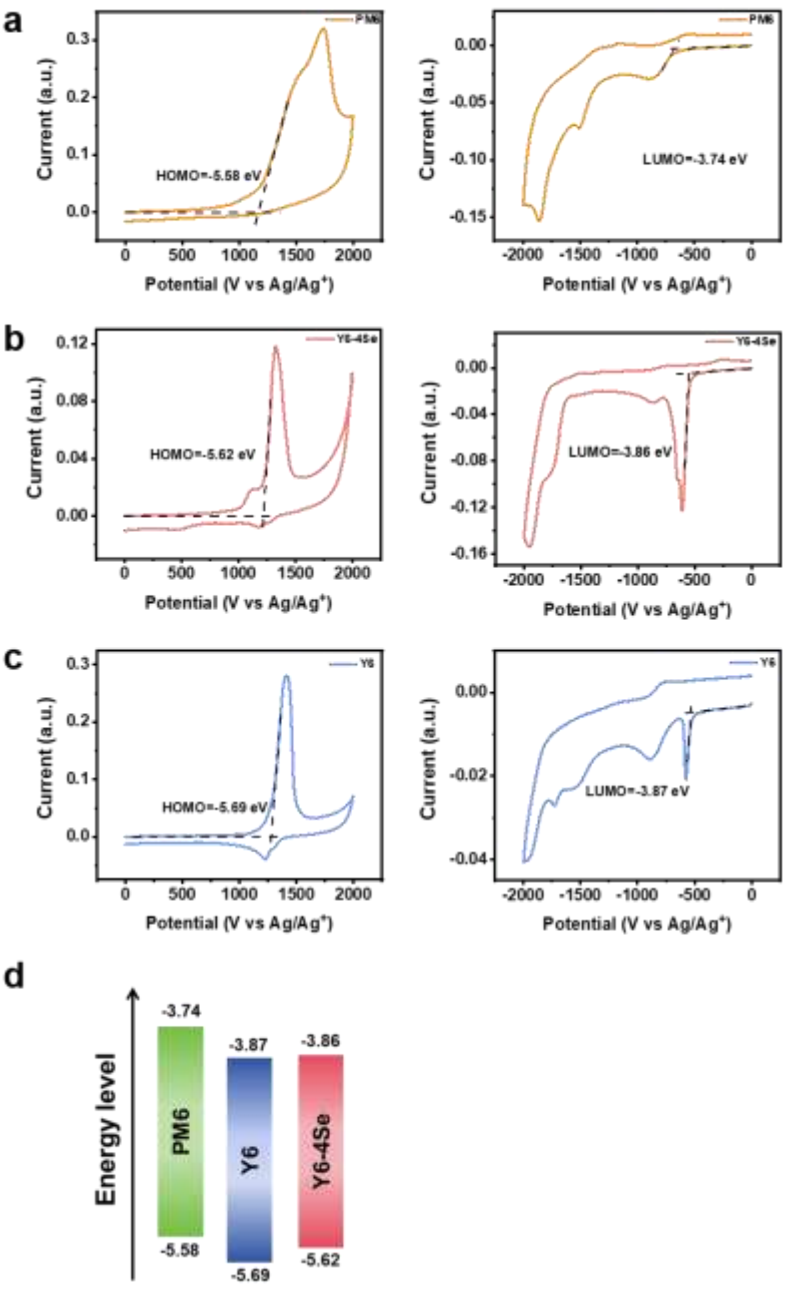

Figure S2. Optoelectronic characterizations of OPDs. Cyclic voltammetry plots of (a) PM6, (b) 4Se, and (c) Y6 films, respectively. Their chloroform solutions were deposited on the surface of the working electrode to form their corresponding films, respectively. (d) The energy level diagram of PM6, Y6, and Y6-4Se.

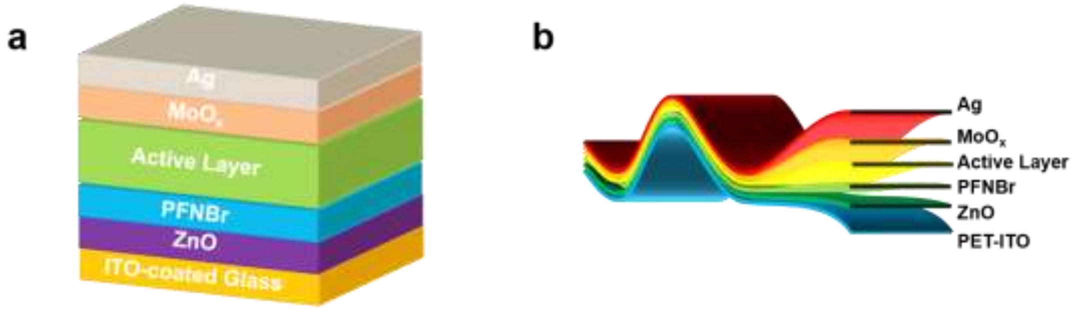

Figure S3. OPD device structure. Device structure of rigid (a) and flexible (b) OPDs.

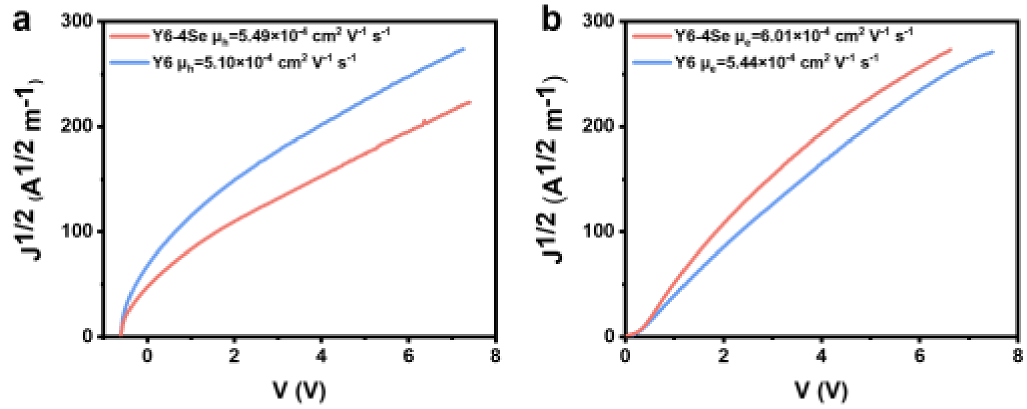

Figure S4. Measurement of charge carrier mobilities. (a) Hole mobilities, (b) electron mobilities of devices based on PM6:Y6-4Se and PM6:Y6 blends.

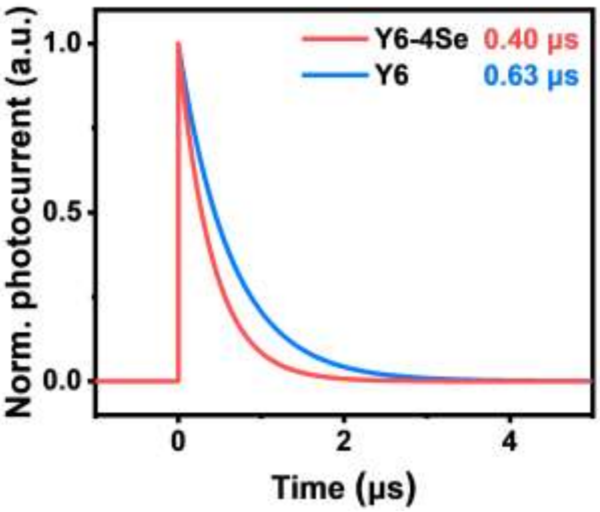

Figure S5. Transient photocurrent measurements of devices based on PM6:Y6-4Se and PM6:Y6 analyzed by a one-order exponential decline model.

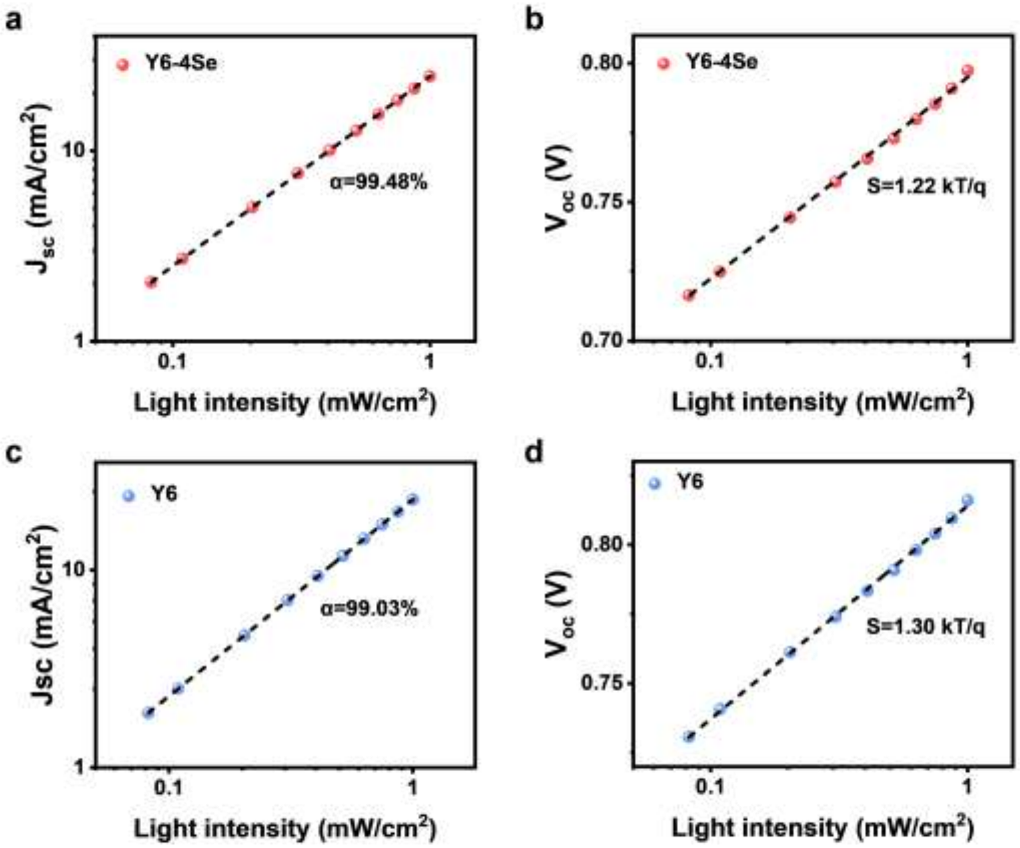

Figure S6. Variation of $\boldsymbol{V_{oc}}$ and $\boldsymbol{J_{sc}}$ with illumination intensity for the OPDs based on PM6-Y6-4Se and PM6:Y6. Dependences of J_{sc} on P_{light} for devices based on PM6-Y6-4Se (a) and PM6:Y6 (c), and those of V_{oc} on P_{light} for devices based on PM6-Y6-4Se (b) and PM6:Y6 (d).

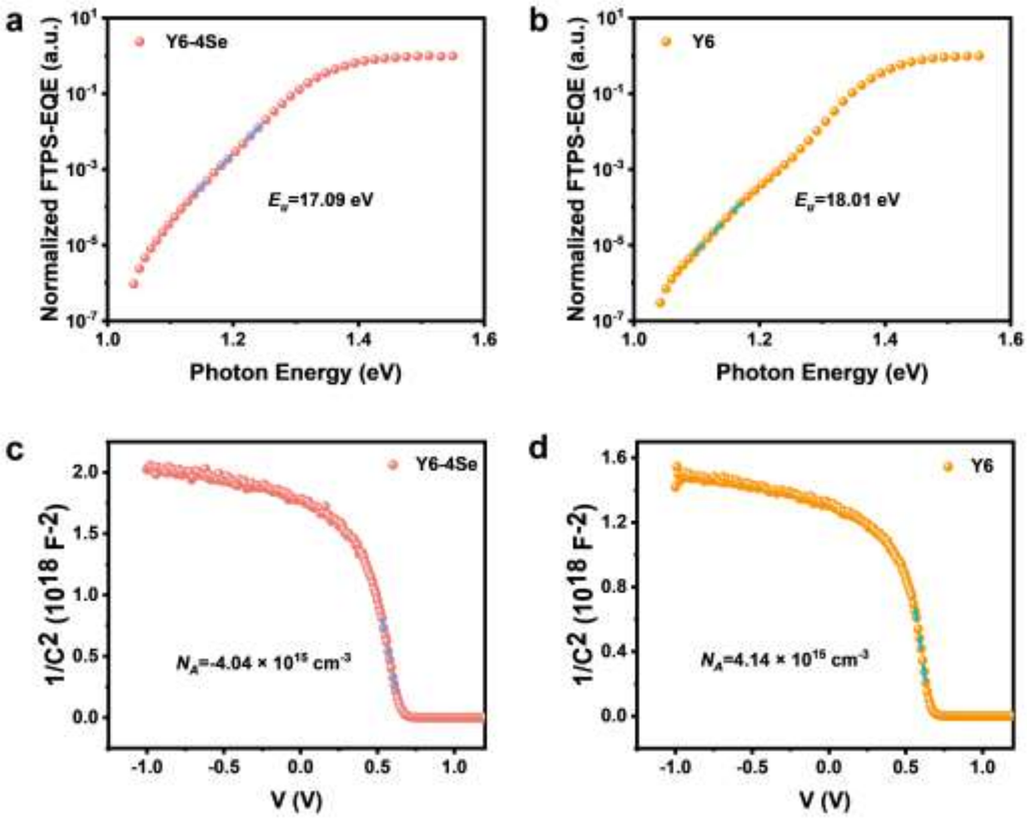

Figure S7. Measurement of Capacitance Spectra and FTPS-EQE. Normalized FTPS-EQE of OPDs prepared with (a) PM6:Y6-4Se and (b) PM6:Y6. Mott-Shockley plots of optimal (c) PM6:Y6-4Se-based and (d) PM6:Y6-based OPDs (dashed lines represent the linear fitting).

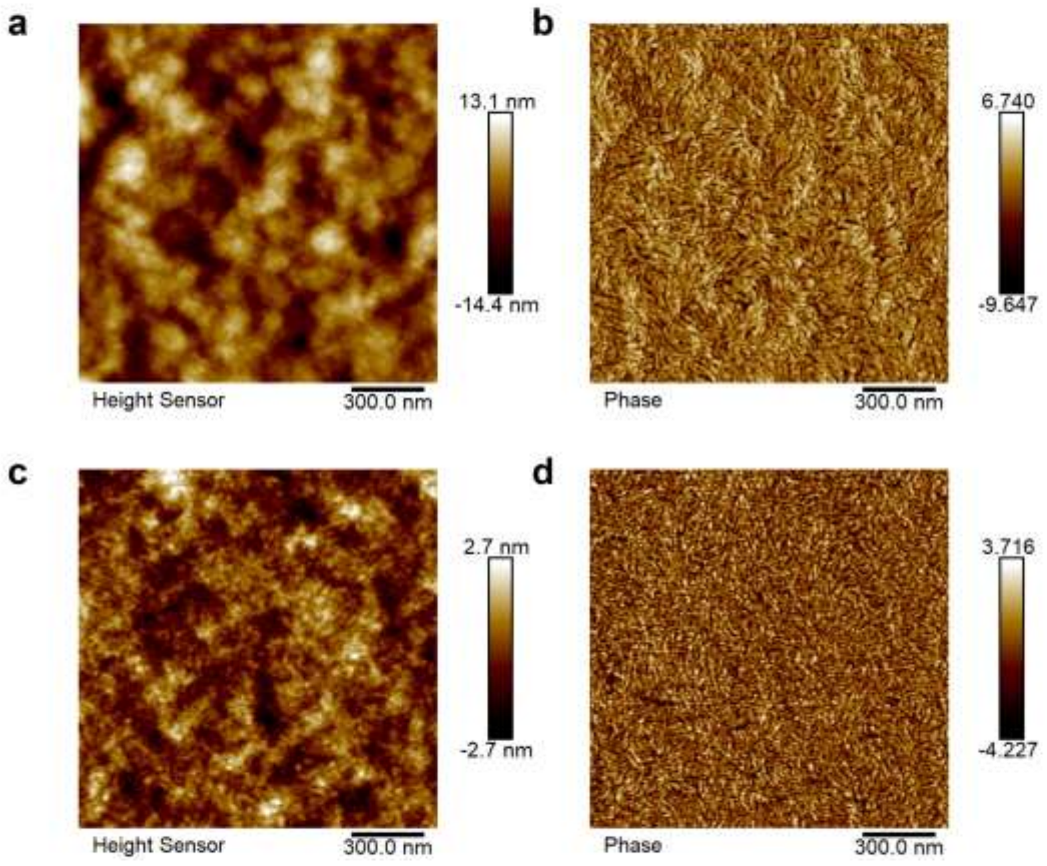

Figure S8. Measurement of AFM. AFM height images of PM6:Y6-4Se (a) and PM6:Y6 (c). AFM phase images of PM6:Y6-4Se (b) and PM6: Y6 (d).

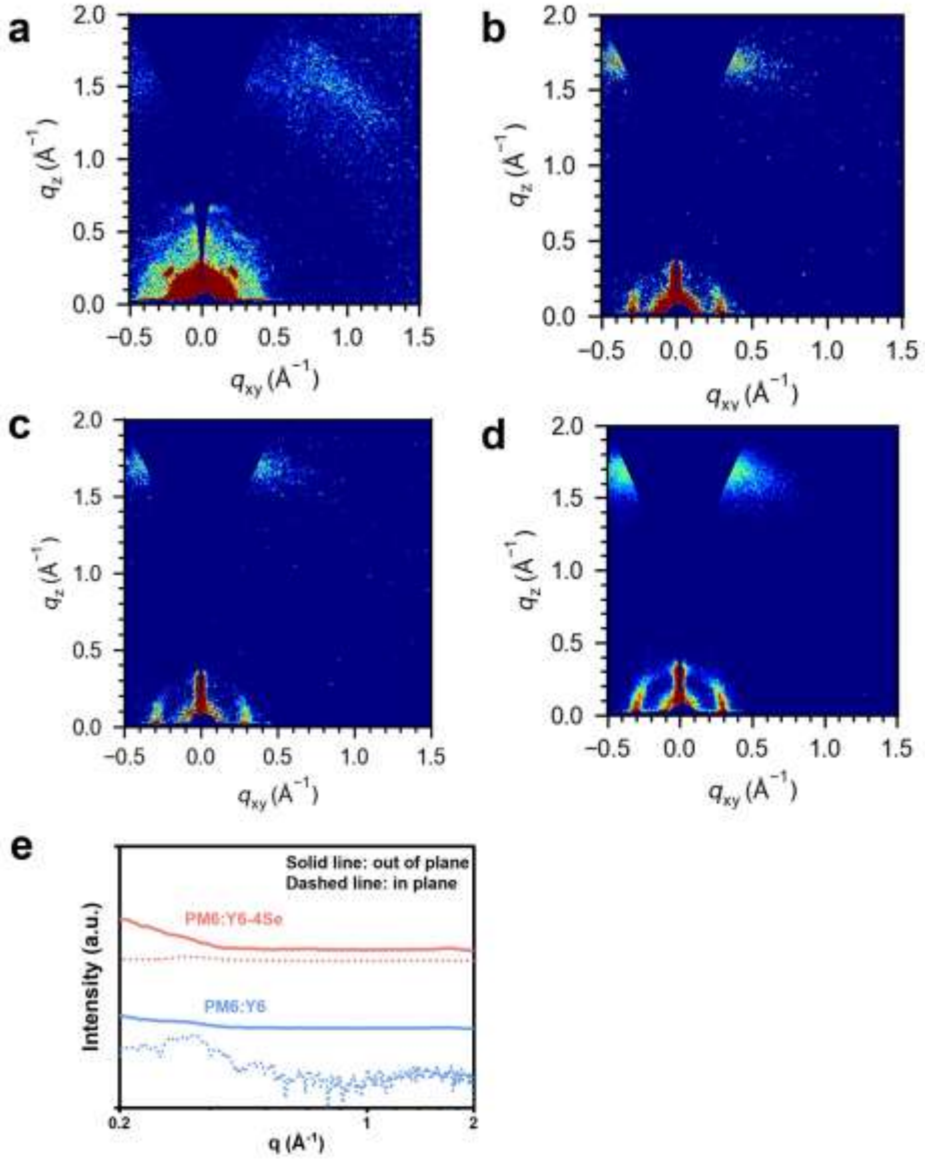

Figure S9. Measurement of GIWAX. 2D GIWAXS patterns of (a) Y6, (b) Y6-4Se neat films and (c) PM6:Y6, (d) PM6:Y6-4Se blends and the corresponding 1D line-cuts (e).

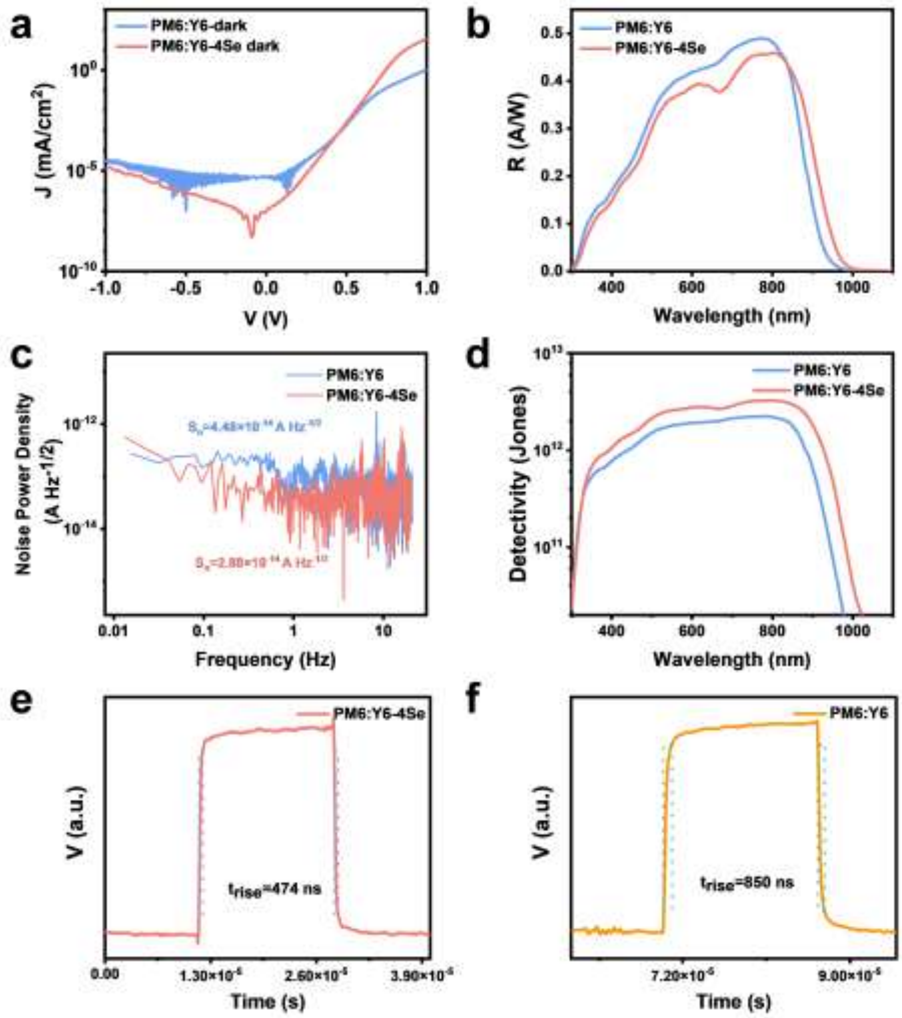

Figure S10. The optoelectronic performance of OPDs based on PM6:Y6-4Se and PM6:Y6. (a) The current density-voltage (J-V) characteristics of the rigid device based on PM6:Y6-4Se (Red Curve) and PM6:Y6 (Blue Curve) blends in the dark. (b) Responsivity; (c) noise current spectrum; and (d) specific detectivity of OPDs in self-powered mode. (e)-(f)**,** The response time of devices based on PM6:Y6-4Se and PM6:Y6 blends in self-powered mode.

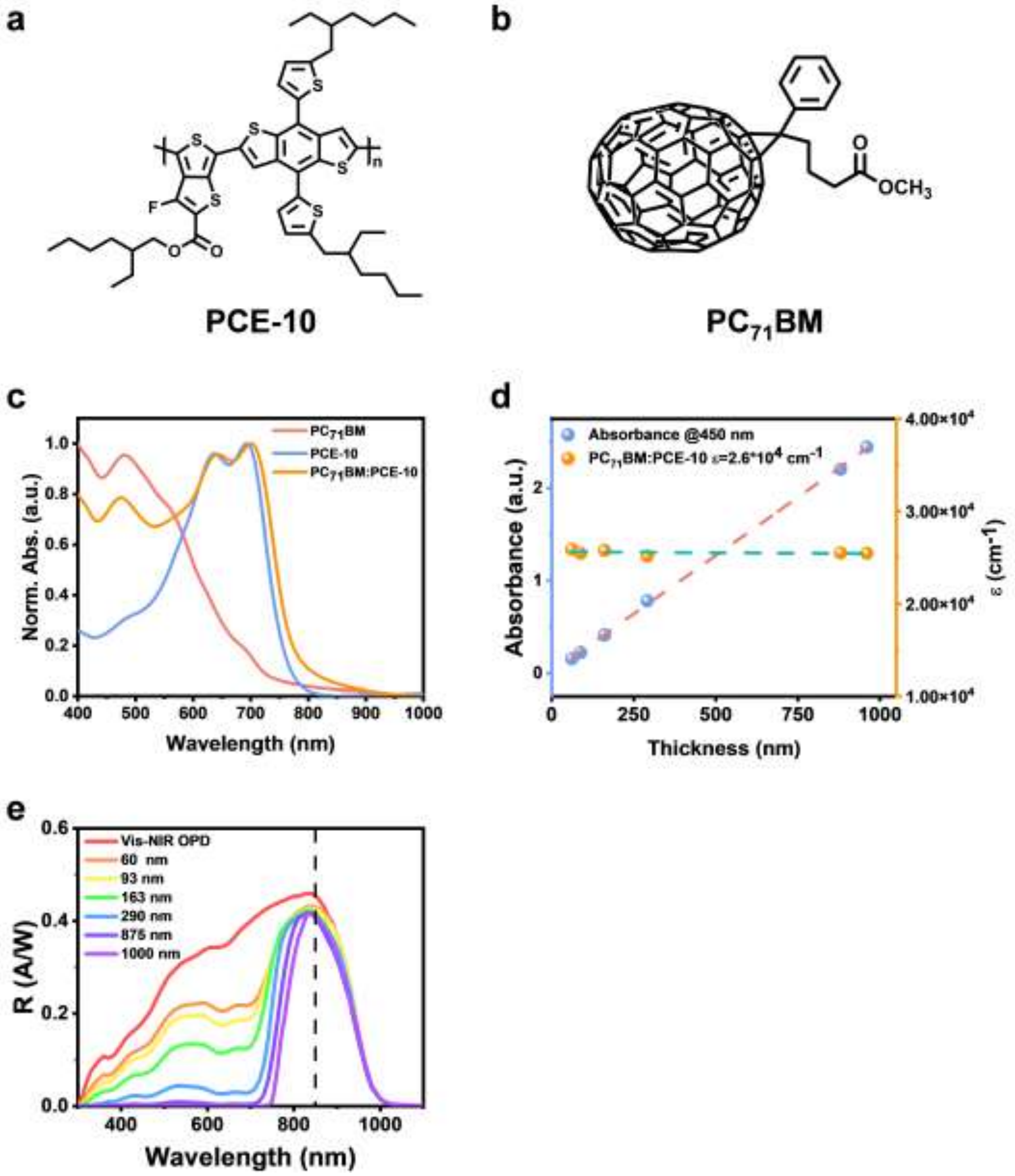

Figure S11. Chemical structures and the absorption profile of the OAL materials. The chemical structures of PCE-10 (a) and PCBM (b) and the absorption spectrum of PCE-10 (Blue Curve), $PC_{71}BM$ (Red Curve), and their blend film (Orange Curve) (c). (d) The absorbance and molar extinction coefficient for OALs with different thicknesses. (e) The responsivities of Vis-blind NIR OPDs with various thicknesses of OALs.

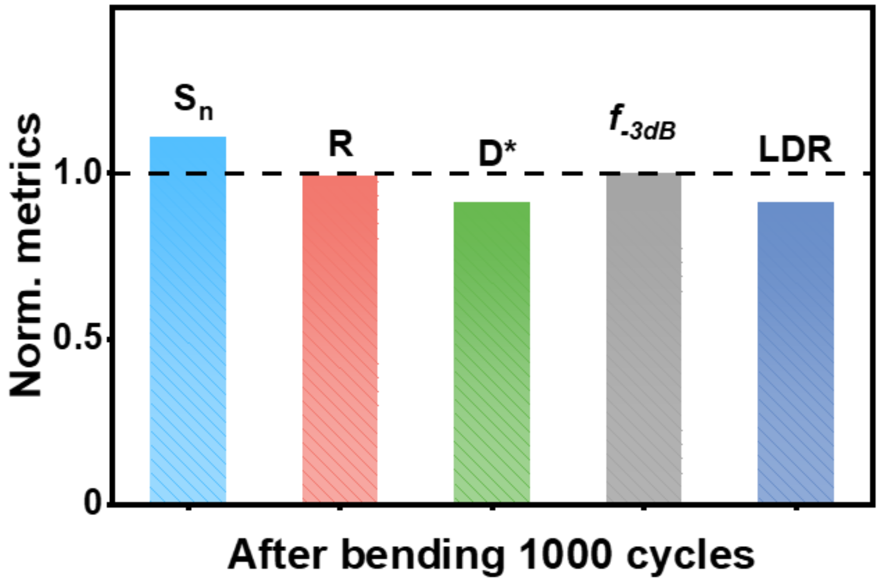

Figure S12. The variation of the Vis-blind NIR OPD's performance parameters after a thousand bending cycles.

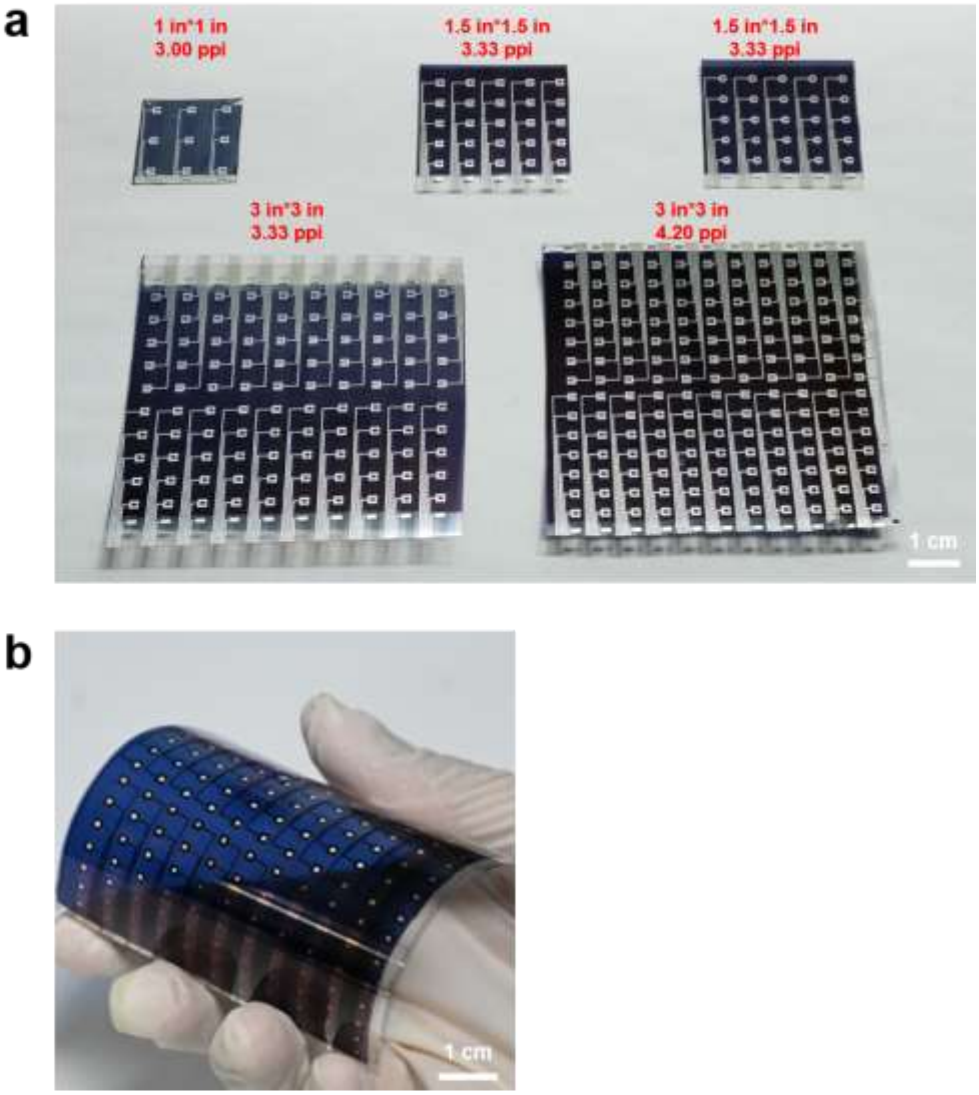

Figure S13. Large-area fabrication of Flex-PCIs. (a) Optical images of Flex-PCIs with various areas, element shapes, and resolutions. (b) Optical image of large-area Flex-PCI in bending condition.

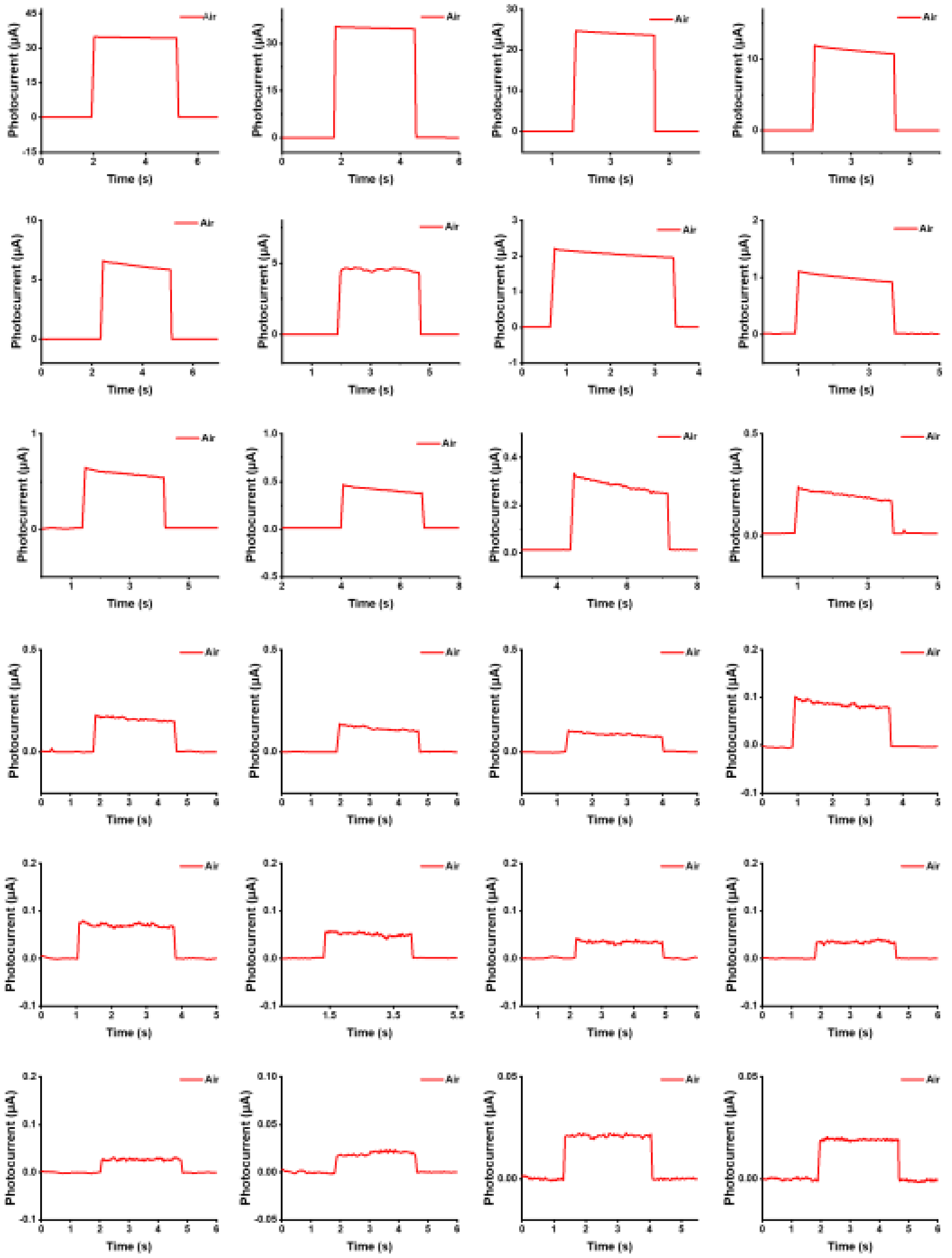

Figure S14. Raw data for measuring the working distance of Flex-PCI in ambient air.

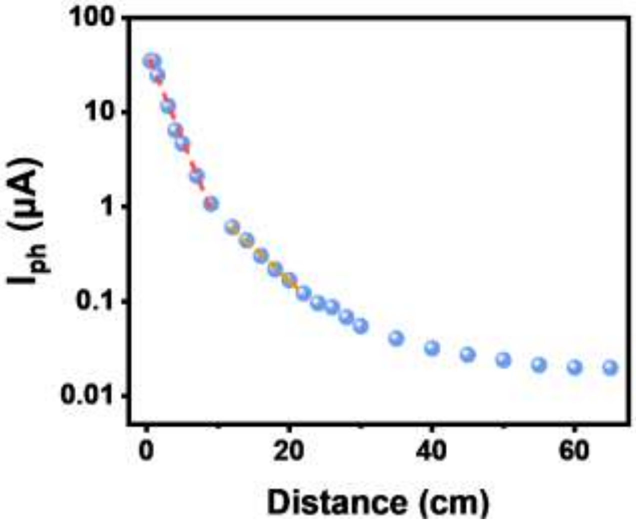

Figure S15. Linear fitting of the logarithm of photocurrent as a function of distance over the ranges of 1.0–10.0 cm and 10.0–22.0 cm.

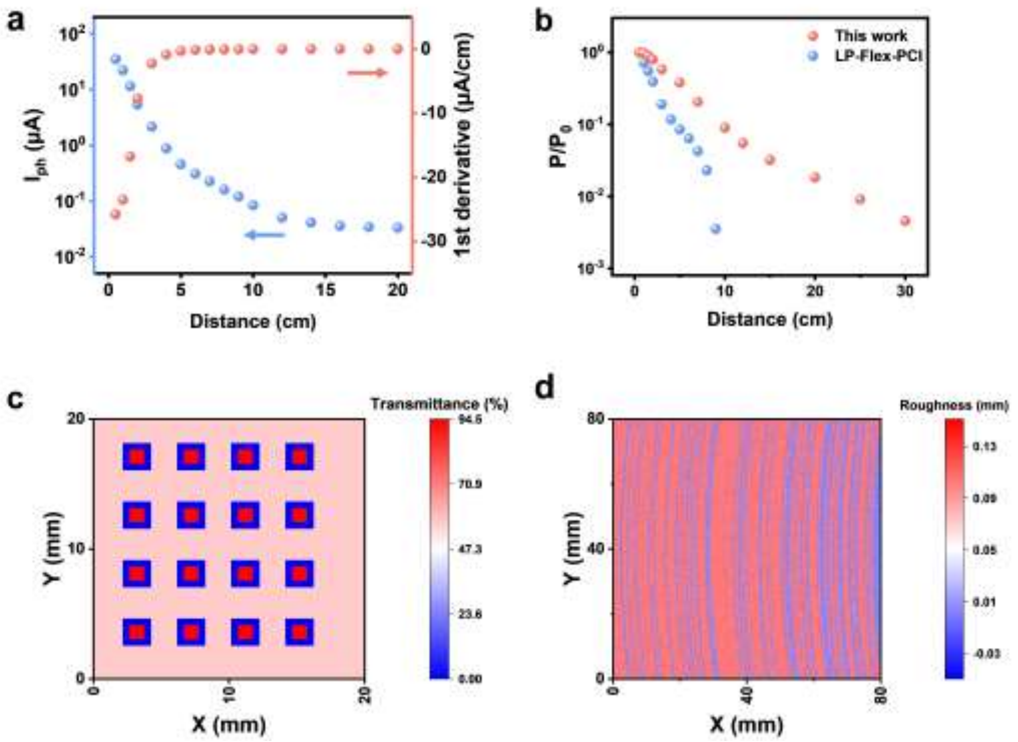

Figure S16. The detection range of LP-Flex-PCI and the optical simulation of the working range. (a) Response behavior of one pixel in PL-Flex-PCI to the approach of a hand palm and its first derivative from 0.5 to 18.0 cm. The light intensity of the LEDs is 10.7 mW cm^{-2}. (b) The simulated variation in light intensity on the device as a function of the distance from the reflected object for the Flex-PCI and LP-Flex-PCI structures. (c) Transmittance mapping of the device for optical simulation. (d) Surface model of the simulated palm.

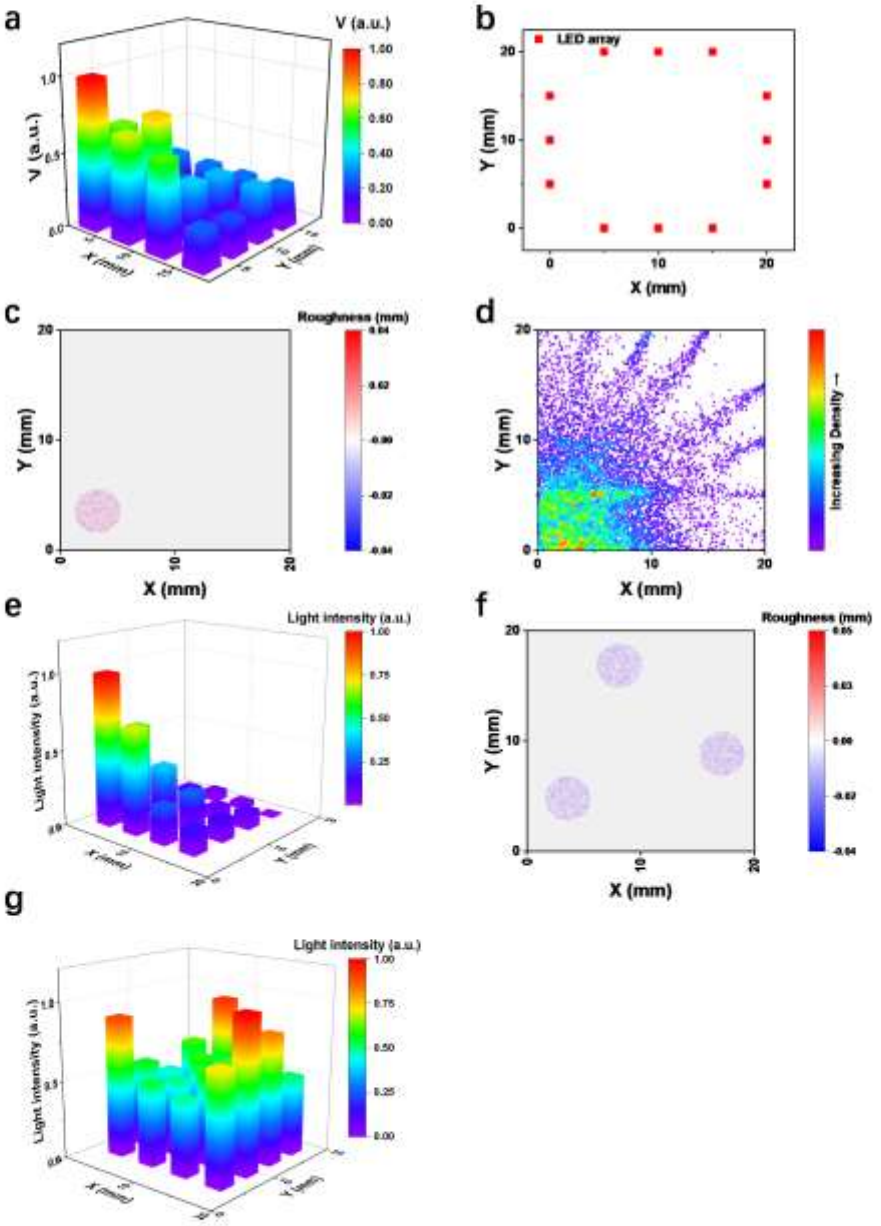

Figure S17. The optical crosstalk simulation of LP-Flex-PCI. (a) Photoresponse of the LP-Flex-PCI in the single-position mode. (b) The position of LEDs in the LP-Flex-PCI device. (c) Surface model of the simulated metal rod for LP-Flex-PCI structure. (d) Simulated ray density distribution for LP-Flex-PCI structure in single-position mode. (e) Simulated light intensity on the pixels of the LP-Flex-PCI device in single-position mode. (f) Surface model of three simulated metal rods for LP-Flex-PCI structure. (g) Simulated light intensity on the pixels of LP-Flex-PCI in multi-position mode.

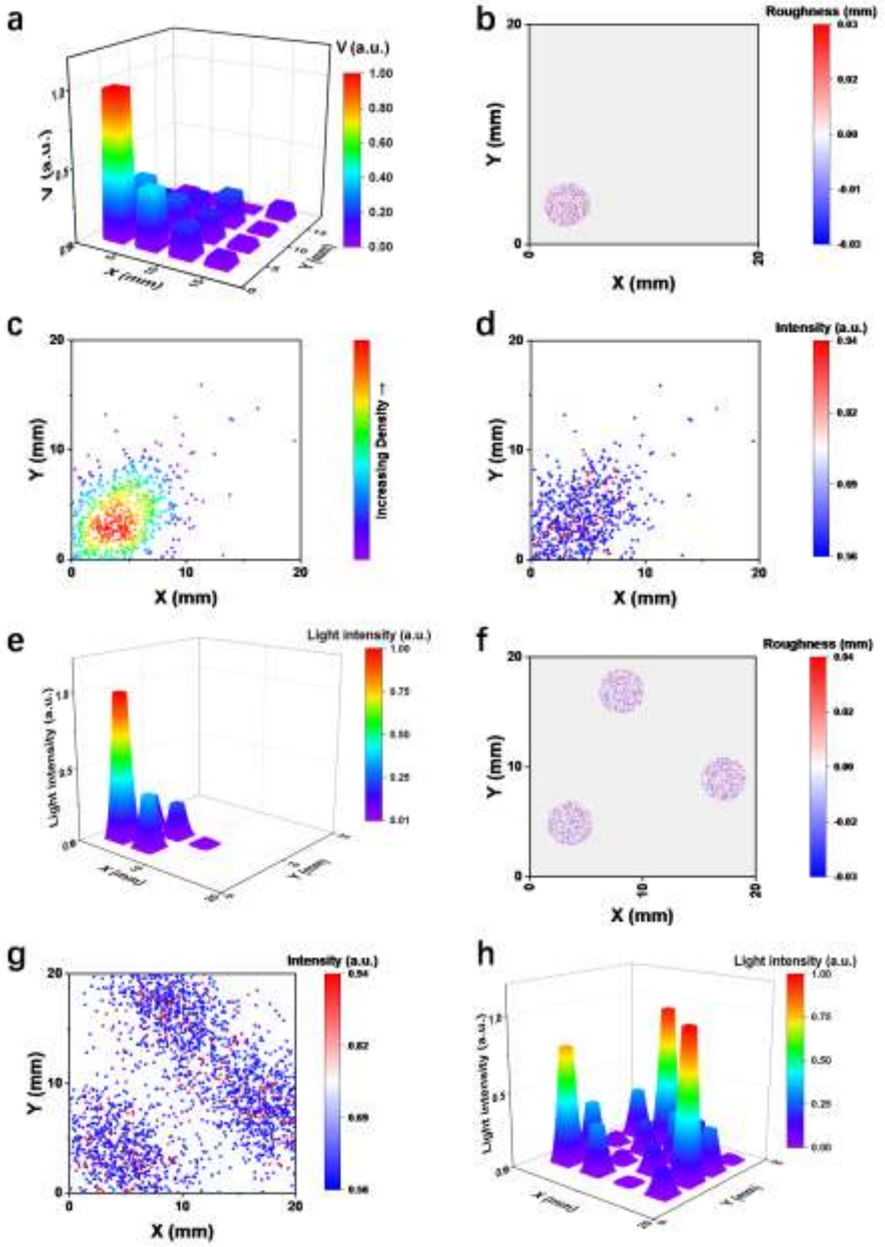

Figure S18. The optical crosstalk simulation of Flex-PCI. (a) Photoresponse of the Flex-PCI in the single-position mode. (b) Surface model of the simulated metal stick. (c) Simulated ray density distribution for Flex-PCI structure in single-position mode. (d) Simulated light intensity distribution for Flex-PCI structure in single-position mode. (e) Simulated light intensity on the pixels of the Flex-PCI device in single-position mode. (f) Surface model of three simulated metal rods. (g) Simulated light intensity distribution for Flex-PCI structure in multi-position mode. (h) Simulated light intensity on the pixels of Flex-PCI in multi-position mode.

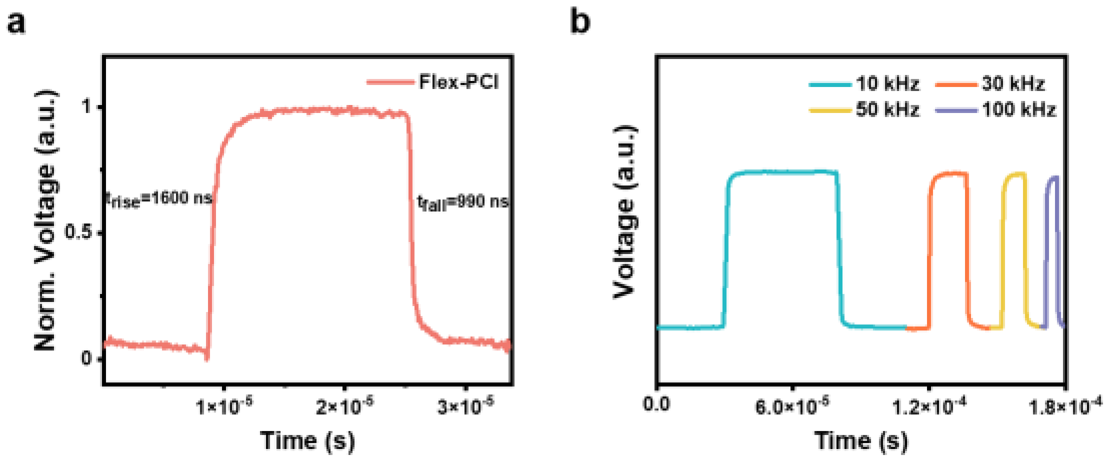

Figure S19. Phototransient performance of the Flex-PCI. (a) Practical response time of Flex-PCI. (b) Phototransient response of the device under light modulation frequencies of 10, 30, 50, and 100 kHz at 0 V.

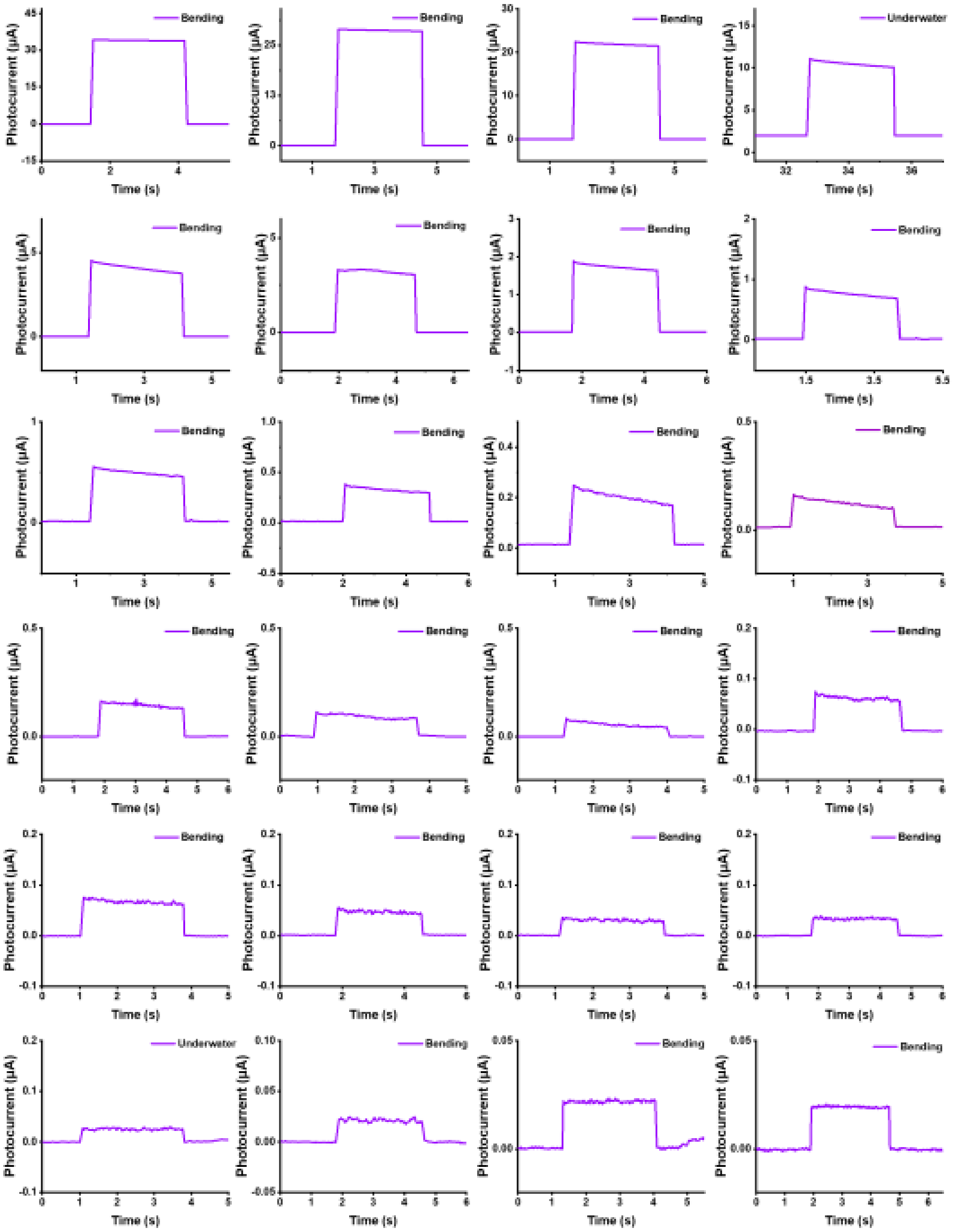

Figure S20. Raw data for measuring the working distance of Flex-PCI under the bending state.

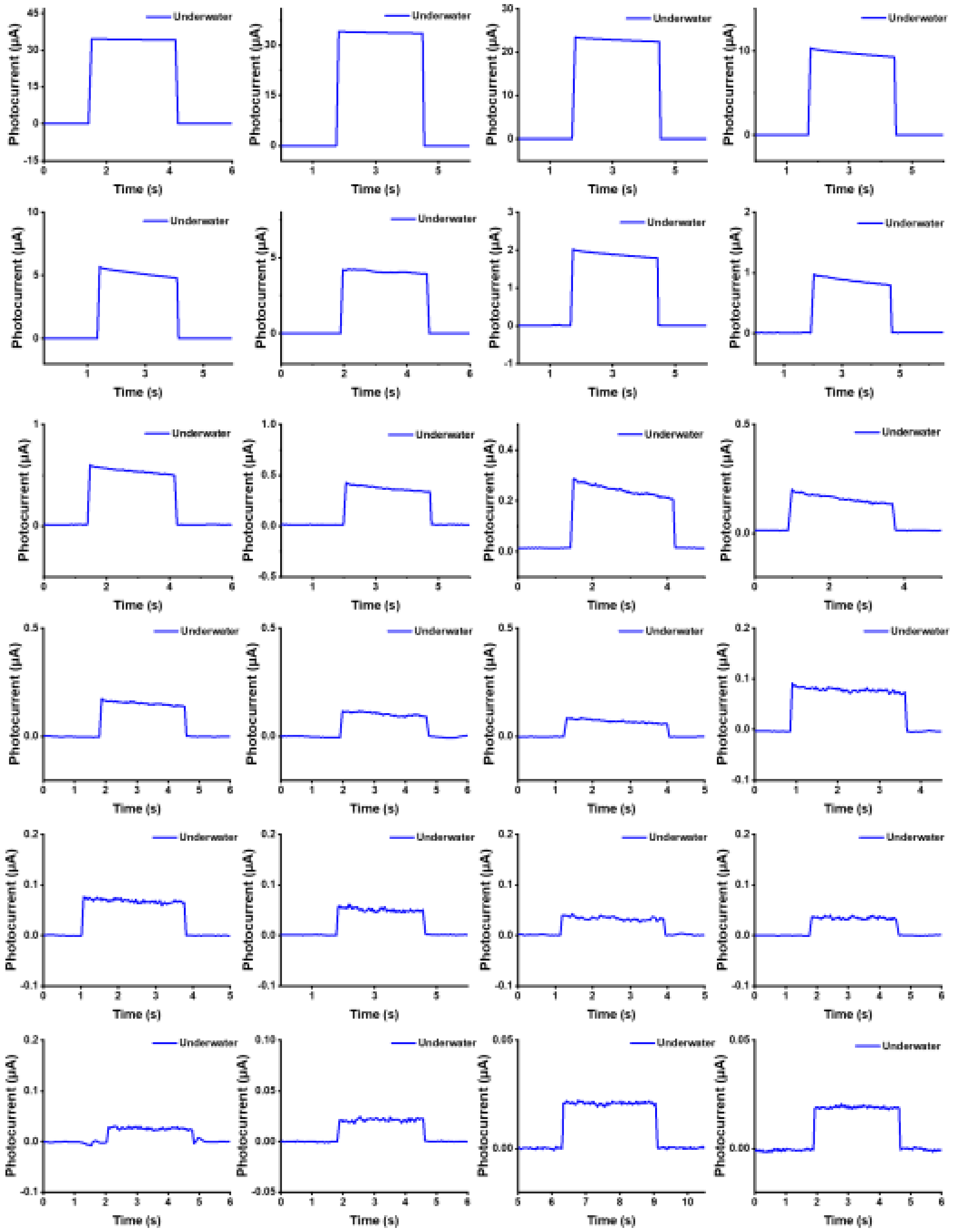

Figure S21. Raw data for measuring the underwater working distance of Flex-PCI.

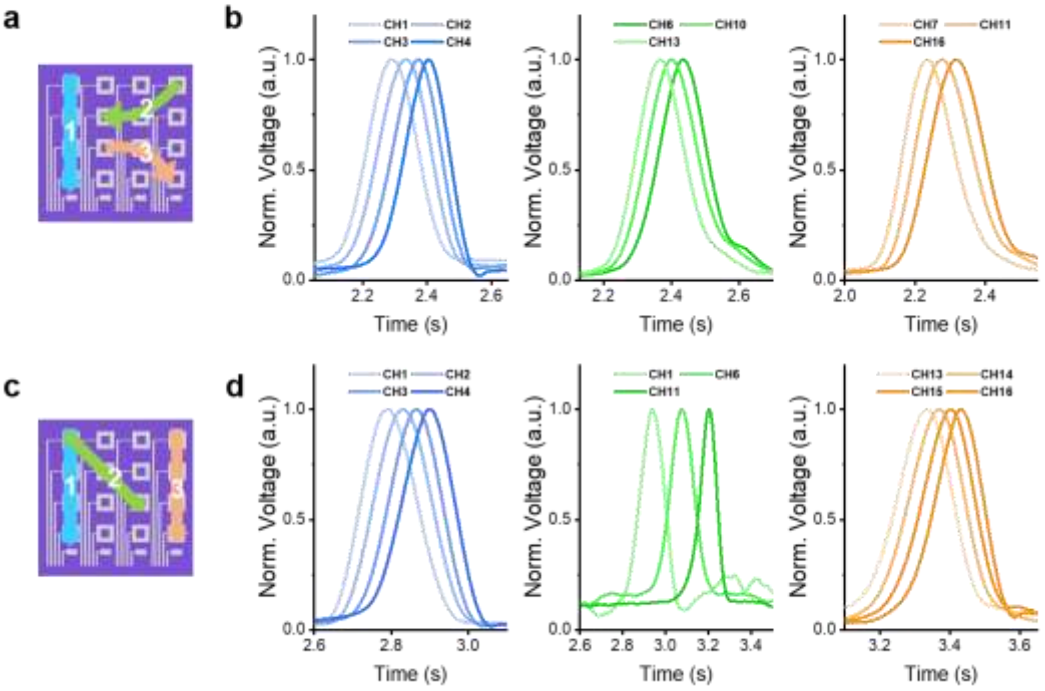

Figure S22. Gesture recognition of Flex-PCI. The schematic diagram for English letters “K” (a) and “N” (c) recognition achieved via mapping trigger sequences to sign language. Arrows inside the diagram depict schematic trigger sequences based on handwriting order. Multichannel voltage outputs of the Flex-PCI when recognizing an English letter, (b) “K”, and (d) “N”.

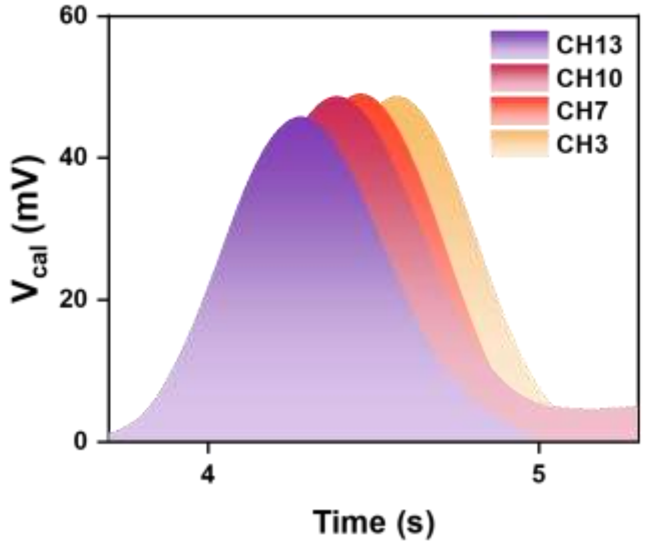

Figure S23. V_{cal} for 4D password system. V_{cal} is the photovoltage of pixels in the Flex-PCI when a metal stick swipes over the device at a distance of 1 cm.

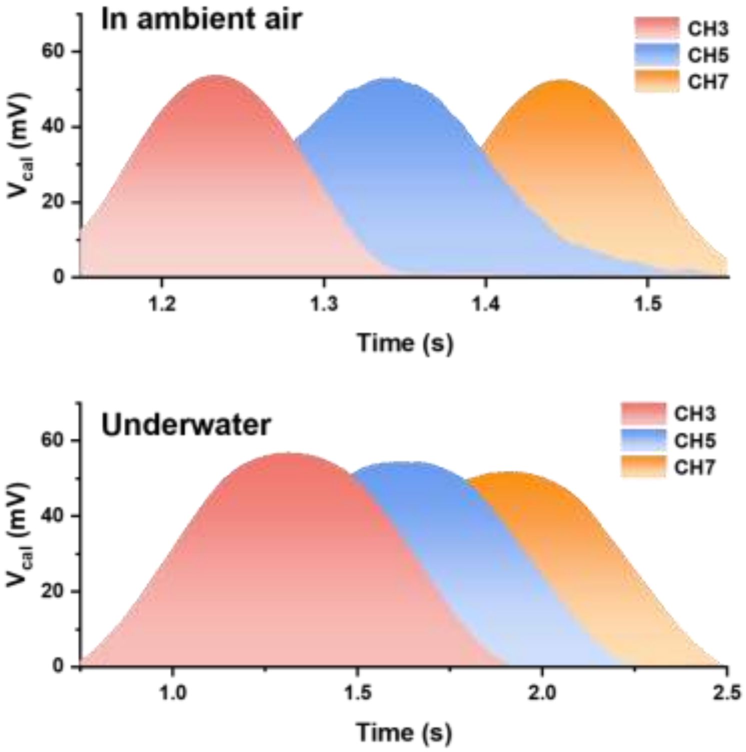

Figure S24. V_{cal} in ambient air and underwater. V_{cal} is the photovoltage of pixels in the Flex-PCI when a finger swipes over the device at a distance of 1 cm.

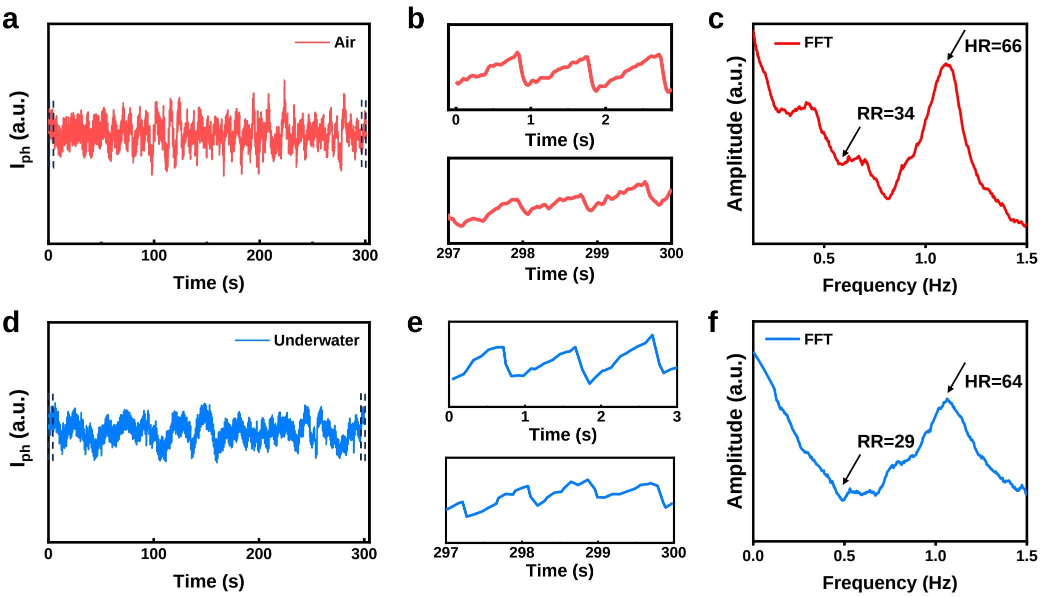

Figure S25. The long-time period pulse waveform measurement. The pulse waveform contactlessly measured using the Flex-PCI by fixing a finger over the device at 1 cm in ambient air (a) and underwater (d) for 5 min. The enlarged views of the pulse waveform in the first and the last 3 s measured in ambient air (b) and underwater (e). The FFT analysis results of the 5-minute pulse waveforms in ambient air (c**)** and underwater (f).

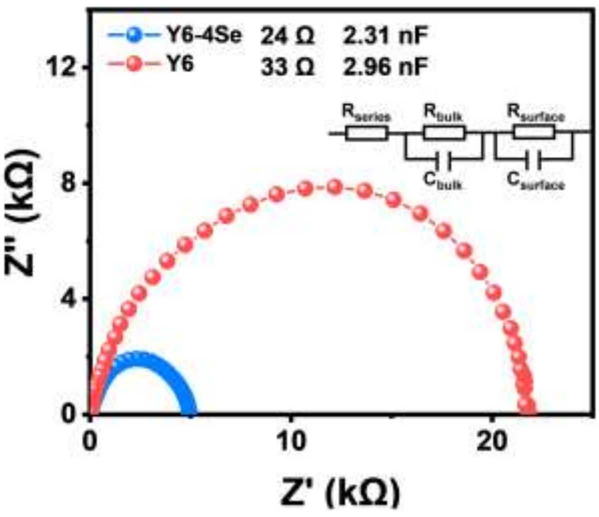

Figure S26. Nyquist plots of devices based on PM6:Y6-4Se and PM6:Y6. Inset: The equivalent-circuit model employed for fitting of EIS data.

Supplementary Tables

Table S1. Performance summary of literature-reported C-HMIs.

Mechanism	Response/recovery Time	Detection Range	Stability	Function	Ref
Humidity	1s/3s	8 mm	800 cycles	2	[23]
Humidity	0.4s/2s	<10 mm	1000 cycles	2	[24]
Triboelectric	-	10 cm	-	2	[25]
Humidity	1.13 s	8 mm	-	2	[26]
Magnetic	-	~ cm level	400 cycles	1	[27]
Triboelectric	-	20 cm	10000 cycles	1	[28]
Magnetic	0.03s	-	100 cycles	1	[29]
Triboelectric	10 ms	25 mm	1000 cycles	1	[30]
Optical	2.4 ms/2.8 ms	60 mm	-	1	[31]
Thermal radiation	0.97 s	2.5 mm	200 compressions	1	[32]
Triboelectric	ms level	25 mm	500 cycles	1	[33]
Triboelectric	5 Hz	14 cm	10000 cycles	1	[34]
Triboelectric	120 ms	20 mm	1200 cycles	1	[35]
Humidity	8 s	20 mm	-	2	[36]
Thermal radiation	0.47 s/0.47 s	10 mm	7 months in air	1	[37]
**Optical**	**1600 ns/990 ns**	**60 cm**	**45000 cycles**	**4**	**Our work**

Table S2. Summary of energy levels and spectral absorption properties of donors and acceptors.

Molecule	E_{HOMO} (eV)	E_{LUMO} (eV)	$\lambda_{sol\ Max}$ (nm)	$\lambda_{Film\ Max}$ (nm)	$\lambda_{abs\ Onset}$ (nm)
PM6	-5.58	-3.74	614	616	686
Y6-4Se	-5.62	-3.86	766	850	946
Y6	-5.69	-3.87	731	817	920

Table S3. The parameters of the space-charge limited current (SCLC).

**Active layer**	**μ_e of blend film ($\times 10^{-4}$ cm^2 V^{-1} s^{-1})**	**μ_h of blend film ($\times 10^{-4}$ cm^2 V^{-1} s^{-1})**	**μ_e/μ_h**
Y6	5.44	5.10	1.07
Y6-4Se	6.01	5.49	1.09

Table S4. The parameters of OPDs calculated via Mott-Shockley analysis.

Active layer	Slope (F^{-2} V^{-1})	N_A (cm^{-3})	V_{bi} (V)	W at 0 V (nm)
Y6	-6.44×10^{18}	4.14×10^{15}	0.66	230.29
Y6-4Se	-6.60×10^{18}	4.04×10^{15}	0.65	231.35

Table S5. Summary of E_u for OPDs based on PM6:Y6-4Se and PM6:Y6.

**Active layer**	**E_u (meV)**
PM6:Y6	18.01
PM6:Y6-4Se	17.09

Table S6. The detailed information from GIWAXS.

	PM6: Y6	PM6: 4Se
**CCL (Å)**	27.05	29.49
**FWHM ($Å^{-1}$)**	0.20	0.19
**q ($Å^{-1}$)**	1.65	1.68
**d (Å)**	3.81	3.74
	oop	

Table S7. The figure of merits of rigid and flexible OPDs.

Active layer	Device	Response time (ns)	R (A W^{-1}) at 850 nm	Sn (A $Hz^{-1/2}$)	D* (Jones) at 850 nm
PM6:Y6	Rigid	850	0.39	4.48×10^{-14}	1.08×10^{12}
PM6:Y6-4Se	Rigid	474	0.43	2.88×10^{-14}	3.07×10^{12}
	Flexible Vis-blind NIR OPD	550	0.34	3.40×10^{-14}	1.97×10^{12}

Table S8. Performance summary of literature-reported flexible NIR OPDs.

Active layer	Response time	Recovery time	f_{-3dB}	Wavelength	Bias (V)	Ref.
PCE-10:F8IC	640 ns	650 ns	-	905 nm	0	[38]
PM6/Y6:PCBM	<200 ms	-	-	405 nm	0	[39]
BDP-Ome:C60	62 μs	27 μs	75.2 kHz	730 nm	0	[40]
D18-Cl:Y6	81 μs	77 μs	8.2 kHz	Green light 530 nm	0	[41]
PCE-10:YZ1	1.4 μs	600 ns	-	905 nm	0	[7]
BDTT-TR:PC61BM	1.4 ms	0.3 ms	-	528 nm	5	[42]
P3HT:IDTBR	-	-	1 MHz	760 nm	-2V	[43]
D18:BTP-4F	9.3 μs	8.7 μs	33 kHz	800 nm	0	[44]
P3HT:PCBM	-	-	70 Hz	Red light 670 nm	-4V/-2V	[45]
PTB7-Th:COTIC-4F:$PC_{71}BM$	-	-	0.95 MHz (Binary) 1.14MHz (Ternary)	940 nm	0	[46]
PTB7-Th:IEICO-4F	-	-	300 kHz	532 nm	0	[47]
PM6:CH17	189.5 ns	175.9 ns	-	880 nm	0	[48]
**PM6:Y6-4Se**	**550 ns**	**460 ns**	**1.32 MHz**	**850 nm**	**0**	**Our work**

References

1. Song G, Feng W, Li Y *et al.* Extending Se substitution to the limit: from 5S to 5Se in high-efficiency non-fullerene acceptors. *Chem Commun* 2023; **59**: 10307-10.

2. Lin F, Zuo L, Gao K *et al.* Regio-specific selenium substitution in non-fullerene acceptors for efficient organic solar cells. *Chem Mater* 2019; **31**: 6770-8.

3. Fan B, Lin F, Wu X *et al.* Selenium-containing organic photovoltaic materials. *Acc Chem Res* 2021; **54**: 3906-16.

4. Wu Z, Li N, Eedugurala N *et al.* Noise and detectivity limits in organic shortwave infrared photodiodes with low disorder. *Npj Flex Electron* 2020; **4**.

5. Ma X, Bin H, van Gorkom BT *et al.* Identification of the origin of ultralow dark currents in organic photodiodes. *Adv Mater* 2022; **35**: 2209598.

6. Kublitski J, Hofacker A, Boroujeni BK *et al.* Reverse dark current in organic photodetectors and the major role of traps as source of noise. *Nat Commun* 2021; **12**: 551.

7. Xia Y, Geng C, Bi X *et al.* Biomimetic flexible high‐sensitivity near‐infrared II organic photodetector for photon detection and imaging. *Adv Opt Mater* 2023; **12**: 2301518.

8. Shockley W, Read WT. Statistics of the recombinations of holes and electrons. *Physical Review* 1952; **87**: 835-42.

9. Kuik M, Koster LJA, Wetzelaer GAH *et al.* Trap-assisted recombination in disordered organic semiconductors. *Phys Rev Lett* 2011; **107**: 256805.

10. Zarrabi N, Sandberg OJ, Zeiske S *et al.* Charge-generating mid-gap trap states define the thermodynamic limit of organic photovoltaic devices. *Nat Commun* 2020; **11**: 5567.

11. Simone G, Dyson MJ, Weijtens CHL *et al.* On the origin of dark current in organic photodiodes. *Adv Opt Mater* 2019; **8**: 1901568.

12. Coropceanu V, Cornil J, da Silva Filho DA *et al.* Charge transport in organic semiconductors. *Chem Rev* 2007; **107**: 926-52.

13. Kaiser C, Sandberg OJ, Zarrabi N *et al.* A universal Urbach rule for disordered

organic semiconductors. *Nat Commun* 2021; **12**: 3988.
14. Scaccabarozzi AD, Basu A, Aniés F *et al.* Doping approaches for organic semiconductors. *Chem Rev* 2021; **122**: 4420-92.
15. Zhang C, Song A, Huang Q *et al.* All-polymer solar cells and photodetectors with improved stability enabled by terpolymers containing antioxidant side chains. *Nano-Micro Lett* 2023; **15**: 140.
16. Li Z, Peng F, Ying L *et al.* Fine tuning miscibility of donor/acceptor through solid additives enables all-polymer solar cells with 15.6% efficiency. *Solar RRL* 2021; **5**: 2100549.
17. Kyaw AKK, Wang DH, Gupta V *et al.* Intensity dependence of current–voltage characteristics and recombination in high-efficiency solution-processed small-molecule solar cells. *ACS Nano* 2013; **7**: 4569-77.
18. Xu Y, Lin Q. Photodetectors based on solution-processable semiconductors: Recent advances and perspectives. *Appl Phys Rev* 2020; **7**: 011315.
19. Li Y, Jiang X, Chen Y *et al.* A platform for integrated spectrometers based on solution-processable semiconductors. *Light Sci Appl* 2023; **12**: 184.
20. Quan H, Zhong Z, Zhou Z *et al.* High-efficiency semitransparent near-infrared organic photodetectors enabled by a molecular crystal network. *Adv Opt Mater* 2024; **12**: 2400818.
21. Jansen-van Vuuren RD, Armin A, Pandey AK *et al.* Organic photodiodes: The future of full color detection and image sensing. *Adv Mater* 2016; **28**: 4766-802.
22. Jiang B-H, Hsiao F-C, Lin Y-R *et al.* Highly efficient ternary near-infrared organic photodetectors for biometric monitoring. *ACS Appl Mater Interfaces* 2023; **15**: 10907-17.
23. Wang J, Lin W, Chen Z *et al.* Smart touchless human-machine interaction based on crystalline porous cages. *Nat Commun* 2024; **15**: 1575.
24. He J, Wei R, Ma X *et al.* Contactless user-interactive sensing display for human-human and human-machine interactions. *Adv Mater* 2024; **36**: 2401931.
25. Dutta A, Niu Z, Abdullah AM *et al.* Closely packed stretchable ultrasound array

fabricated with surface charge engineering for contactless gesture and materials detection. *Adv Sci* 2024: 2303403.

26. Qi M, Xu R, Ding G *et al.* An in-sensor humidity computing system for contactless human-computer interaction. *Mater Horiz* 2024; **11**: 939-48.

27. Jiang C, Xu H, Yang L *et al.* Neuromorphic antennal sensory system. *Nat Commun* 2024; **15**: 2109.

28. Mu S, Li S, Zhao H *et al.* A platypus-inspired electro-mechanosensory finger for remote control and tactile sensing. *Nano Energy* 2023; **116**: 108790.

29. Li S, Wu Y, Asghar W *et al.* Wearable magnetic field sensor with low detection limit and wide operation range for electronic skin applications. *Adv Sci* 2023; **11**: 2304525.

30. Liu W, Duo Y, Chen X *et al.* An intelligent robotic system capable of sensing and describing objects based on bimodal, self‐powered flexible sensors. *Adv Funct Mater* 2023; **33**: 2306368.

31. Kamijo T, van Breemen AJJM, Ma X *et al.* A touchless user interface based on a near-infrared-sensitive transparent optical imager. *Nat Electron* 2023; **6**: 451-61.

32. Gao FL, Min P, Ma Q *et al.* Multifunctional thermoelectric temperature sensor for noncontact information transfer and tactile sensing in human‐machine interaction. *Adv Funct Mater* 2023; **34**: 2309553.

33. Zhou H, Huang W, Xiao Z *et al.* Deep‐learning‐assisted noncontact gesture‐recognition system for touchless human‐machine interfaces. *Adv Funct Mater* 2022; **32**: 2208271.

34. Shrestha K, Sharma S, Pradhan GB *et al.* A siloxene/ecoflex nanocomposite‐based triboelectric nanogenerator with enhanced charge retention by MoS_2/LIG for self‐powered touchless sensor applications. *Adv Funct Mater* 2022; **32**: 2113005.

35. Liu W, Duo Y, Liu J *et al.* Touchless interactive teaching of soft robots through flexible bimodal sensory interfaces. *Nat Commun* 2022; **13**: 5030.

36. Le X, Shi Q, Sun Z *et al.* Noncontact human-machine interface using complementary information fusion based on MEMS and triboelectric sensors. *Adv Sci*

2022; **9**: 2201056.
37. Guo X, Lu X, Jiang P *et al.* $SrTiO_3$/CuNi-heterostructure-based thermopile for sensitive human radiation detection and noncontact human-machine interaction. *Adv Mater* 2022; **34**: 2204355.
38. Zhu Y, Qin H, Guo T *et al.* A universal strategy for narrowband organic photodetectors enabling arbitrary narrow spectrum detection. *Sci China Mater* 2024; **67**: 852-62.
39. Yang G, Zhang D, Wang R *et al.* Flexible broadband organic photodetectors with ternary planar-mixed heterojunction semiconductors and solution-processed polymeric electrode. *ACS Appl Mater Interfaces* 2024; **16**: 23643−53.
40. Wang Y, Zhang T, Samigullina D *et al.* Semitransparent near‐infrared organic photodetectors: Flexible, large‐area, and physical‐vapor‐deposited for versatile advanced optical applications. *Adv Funct Mater* 2024; **34**: 2313689.
41. Schrickx HM, Gyurek S, Moore C *et al.* Flexible self‐powered organic photodetector with high detectivity for continuous on‐plant sensing. *Adv Opt Mater* 2024; **12**: 2400005.
42. Tang Y, Li R, Sun R *et al.* Flexible all-organic photodetectors via universal water-assisted transfer printing. *Innovation* 2023; **4**: 100460.
43. Ruiz‐Preciado LA, Baek S, Strobel N *et al.* Monolithically printed all-organic flexible photosensor active matrix. *Npj Flex Electron* 2023; **7**.
44. Pu K, Xu Z, Gao Y *et al.* A flexible sensitive visible‐NIR organic photodetector with high durability. *Adv Mater Technol* 2023; **8**: 2300207.
45. Luo G, Shi J, Deng W *et al.* Boosting the performance of organic photodetectors with a solution-processed integration circuit toward ubiquitous health monitoring. *Adv Mater* 2023; **35**: 2301020.
46. Lou Z, Tao J, Wei B *et al.* Near-infrared organic photodetectors toward skin-integrated photoplethysmography-electrocardiography multimodal sensing system. *Adv Sci* 2023; **10**: 2304174.
47. Jan J, Zhu J, Ting J *et al.* Flexible blade‐coated optoelectronic devices: dual

functionality via simultaneous deposition. *Adv Funct Mater* 2022; **32**: 2112343.

48. Zhu Y, Chen H, Han R *et al.* High-speed flexible near-infrared organic photodiode for optical communication. *Natl Sci Rev* 2024; **11**: nwad311.
